# HSPA5 promotes YAP/TAZ stability independently of the Hippo pathway and induces proneural-to-mesenchymal transition in glioblastoma

**DOI:** 10.1038/s41419-026-08428-3

**Published:** 2026-02-07

**Authors:** Shikai Gui, Wanli Yu, Zhen Song, Lunshan Peng, Haitao Luo, Kai Huang, Juexian Xiao, Jiabao Xie, Shihao Cai, Shengtao Yuan, Zhennan Tao, Zujue Cheng

**Affiliations:** 1 Department of Neurosurgery, The Second Affiliated Hospital, Jiangxi Medical College, Nanchang University, Nanchang, Jiangxi China; 2 Jiangxi Key Laboratory of Neurological Tumors and Cerebrovascular Diseases, Nanchang University, Nanchang, Jiangxi China; 3 JXHC key Laboratory of Neurological medicine, Nanchang University, Nanchang, Jiangxi China; 4 Institute of Neuroscience, Nanchang University, Nanchang, Jiangxi China; 5 Department of Ultrasound, The Second Affiliated Hospital, Jiangxi Medical College, Nanchang University, Nanchang, Jiangxi China; 6 Jiangsu Key Laboratory of Drug Screening, China Pharmaceutical University, Nanjing, Jiangsu China; 7 China Pharmaceutical University, Nanjing, Jiangsu China; 8 Department of Neurosurgery, Nanjing Drum Tower Hospital, The Affiliated Hospital of Nanjing University Medical School, Nanjing University, Nanjing, Jiangsu China; 9 Neurosurgical Institute, Nanjing University, Nanjing, Jiangsu China; 10 Department of Neurosurgery, Central Hospital of Shangrao City, Shangrao, Jiangxi China

**Keywords:** HIPPO signalling, CNS cancer

## Abstract

The proneural-to-mesenchymal transition (PMT) is a pivotal process in glioblastoma (GBM), driving enhanced tumor aggressiveness, therapeutic resistance, and recurrence. HSPA5, a member of the heat shock protein 70 (HSP70) family, plays a crucial role in regulating and maintaining protein stability and function. Although HSPA5 is a recognized marker of poor prognosis in glioma, its underlying mechanistic function remains poorly defined. Here, we demonstrated that HSPA5 expression is highest in the mesenchymal (MES) subtype of GBM. The overexpression of HSPA5 in proneural (PN) cells induced PMT and promoted malignant phenotypes, whereas its knockdown in MES cells suppressed PMT and attenuated tumorigenicity. We further established that HSPA5 drives PMT by activating the YAP/TAZ pathway in vitro and in vivo. The expression of MES markers CD44 and c-MET was transcriptionally regulated by YAP/TAZ. Mechanistically, HSPA5 interacts directly with YAP/TAZ, disrupting their association with β-TrCP. This protective interaction inhibits the ubiquitination and proteasomal degradation of YAP/TAZ. Furthermore, HSPA5 expression was positively correlated with YAP and TAZ levels across GBM subtypes. Patients with high expression of HSPA5, YAP, and TAZ exhibited significantly poorer overall survival. Collectively, our findings suggested that HSPA5 promotes PMT through the stabilization of YAP/TAZ and identified HSPA5 as a promising therapeutic target for GBM patients.

## Introduction

Glioblastoma (GBM) is the most prevalent primary malignant brain tumor in adults and is essentially incurable [1, 2]. Despite substantial advances in cancer treatment, the prognosis for patients with glioblastoma remains bleak [3, 4]. Based on gene expression profiles, The Cancer Genome Atlas (TCGA) has categorized glioblastoma into distinct molecular subtypes, including proneural (PN), classical (CL), and mesenchymal (MES) [5–7]. The proneural-to-mesenchymal transition (PMT) is considered a driver of GBM progression and recurrence [8–10]. However, the specific molecular mechanisms of PMT in GBM remain incompletely elucidated and warrant further investigation.

The 70 kDa heat shock proteins (HSP70s) comprise 13 homologous molecular chaperones that play diverse roles in cancer development and progression, including in glioblastoma [11, 12]. Heat shock protein family A (HSP70) member 5 (*HSPA5*), also known as GRP78 or Bip, is predominantly localized to the endoplasmic reticulum (ER) [13]. Under various ER stress, HSPA5 relocates to the cell surface, mitochondria, nucleus, or forms complexes with other proteins to perform its functions [14, 15]. Numerous studies have demonstrated that HSPA5 plays a critical role in maintaining protein stability in various cancers, including pancreatic ductal adenocarcinoma, triple-negative breast cancer, colorectal cancer, and glioblastoma [16–19]. However, the specific role of HSPA5 in maintaining protein stability in glioblastoma remains unclear.

The transcriptional coactivators Yes-associated protein (YAP) and transcriptional coactivator with PDZ-binding motif (TAZ, also known as *WWTR1*) are key downstream effectors of the Hippo signaling pathway. Their frequent activation in solid tumors contributes to aggressive malignant phenotypes and is associated with poor patient prognosis [20]. Normally, when the Hippo pathway is active, mammalian Ste20-like kinases 1 and 2 (MST1/2) phosphorylate large tumor suppressors 1 and 2 (LATS1/2), which then phosphorylate YAP/TAZ, resulting in cytoplasmic sequestration by binding to 14-3-3 proteins [21–23]. Phosphorylated YAP/TAZ subsequently bind to β-TrCP, a ubiquitin ligase, leading to their ubiquitination and subsequent proteasome-mediated degradation [24]. Additionally, activated LATS1/2 prevents YAP/TAZ translocation into the nucleus, inhibiting their binding to transcription factors TEAD1-4 [25]. In glioblastoma, YAP and TAZ are overexpressed and function as master regulators of proliferation, metastasis, and mesenchymal differentiation, including the PMT [26–28]. However, the upstream mechanisms driving the overexpression or activation of YAP/TAZ in GBM remain largely unknown.

In the present study, we found high *HSPA5* expression was correlated with GBM progression and poor prognosis. Mechanistically, we demonstrated that HSPA5 directly interacts with YAP/TAZ, and disrupts their association with β-TrCP. This protective binding impedes YAP/TAZ ubiquitination and proteasomal degradation, thereby promoting the PMT. Furthermore, we showed that HSPA5 knockdown inhibits the malignant phenotype of GBM cells in a YAP/TAZ-dependent manner. Collectively, our findings revealed that HSPA5 promotes PMT through the stabilization of YAP/TAZ, and suggested that HSPA5 could serve as a promising therapeutic target for MES subtype of glioblastoma.

## Materials and methods

### Data acquisition

The transcriptomic datasets and clinical information for GBM patients were downloaded from TCGA database (https://portal.gdc.cancer.gov/), which comprises 175 samples. After we excluded unidentified subtype samples, and 44 proneural, 57 classical, and 49 mesenchymal samples were selected for analysis. Additionally, 20 samples were excluded as they represented normal brain tissue or incomplete clinical data. Finally, the 155 samples were subjected to Kaplan–Meier analysis (Tables S1 and S2). The 13 human HSP70 family members analyzed in this study were obtained from previous literature [11], and the corresponding details are provided in Table S3.

### Human tissue samples

Human GBM samples and clinical characteristics were collected from the Department of Neurosurgery at The Affiliated Drum Tower Hospital of Nanjing University Medical School (Nanjing, Jiangsu, China). Normal brain tissues (NBTs) were obtained from patients undergoing decompressive craniotomy for traumatic brain injury and served as the control group. This study was approved by the Institutional Review Board and the Ethics Committee of the Affiliated Drum Tower Hospital of Nanjing University Medical School. The patient tissue samples were collected and processed in accordance with approved guidelines. Informed consent was acquired from each enrolled patient.

### Primary GBM cell and cell lines culture

Primary GBM cells were isolated from post-surgical specimens of GBM patients. Fifty human GBM samples were classified into PN and MES subtypes based on RNA sequencing analysis. For preparation, the specimens were divided into ~2 × 2× 2 mm^3^ pieces using a scalpel, followed by enzymatic digestion with 0.1% trypsin (Invitrogen, USA) and 10 U/ml DNase I (Promega, USA) for 45 min at 37 °C. The red blood cells were lysed using ACK lysis buffer (Beyotime, Shanghai, China), and the cell suspension was triturated by pipetting and passed through a 100-μm cell strainer. The primary cells were incubated with CD44 antibody or SOX2 antibody at 4 °C for 30–60 min, followed by fluorescence-activated cell sorting (FACS) to isolate PN cells (PN20 and PN24) and MES cells (MES50 and MES52). The sorted cells were validated by western blot analysis and authenticated by STR profiling (Genetic Testing Biotechnology). These cells were cultured in DMEM/F12 (Gibco) supplemented with 10% Gibco fetal bovine serum (FBS) (Gibco) and 1% penicillin–streptomycin (P/S) (Gibco).

HEK293T cells from ATCC were cultured in DMEM (Gibco) with 10% FBS and 1% PS. Normal human astrocytes (NHAs) (ScienCell Research Laboratories, CA, USA) were maintained in astrocyte medium (OTWO Technology Company) with 10% FBS and 1% PS. All the cells were seeded into culture dishes and incubated at 37 °C in a humidified incubator with 5% CO_2_. Cells were treated with the proteasomal inhibitor MG132 (Sigma-Aldrich, USA) at a concentration of 20 µM for 8 h to prevent proteasomal-mediated degradation, or with cycloheximide (CHX, Sigma-Aldrich, USA) at a concentration of 50 µg/mL to block new protein synthesis.

### RNA-seq and bioinformatic analysis

Total RNA was extracted from 50 human GBM samples, stable HSPA5 knockdown MES50 cells, and negative control cells using the TRIzol^TM^ reagent (Cat No. 15596026CN, ThermoFisher, MA, USA) following the manufacturer’s instructions. The concentration and quality of the extracted RNA were determined using a NanoDrop spectrophotometer. RNA sequencing was conducted by Nanjing Allwegene Tech Company. After quality control of the output files, the raw data were transformed into fragments per kilobase of transcript per million fragments mapped (FPKM) format. For the 50 clinical GBM specimens, signature scores for PN, MES, and CL subtypes were calculated using the single-sample gene set enrichment analysis (ssGSEA) method. Subsequently, differential expression analysis was performed using the limma package. The screening criteria for differentially expressed gene (DEG) were |log2 fold change (FC) | ≥ 0.5 and *P* < 0.05. KEGG enrichment analysis was implemented using the DAVID (https://davidbioinformatics.nih.gov/tools.jsp) online functional annotation tool. The gene sets for ‘Verhaak glioblastoma mesenchymal’ and ‘Verhaak glioblastoma proneural’ were sourced from the Molecular Signatures Database (MSigDB; http://www.gsea-msigdb.org/gsea/login.jsp). GSEA was performed using GSEA 4.3.2 software with default parameters. FDR < 0.05 and *P* < 0.05 indicate statistical significance.

### Transfection

To generate stable knockdown cells, human *HSPA5* shRNA (sh*HSPA5*#1: 5’-GAGCGCATTGATACTAGAAAT-3’; sh*HSPA5*#2: 5’-GGAACCATCCCGTGGCATAAA-3’), *YAP1* shRNA (sh*YAP1*: 5’-GCCACCAAGCTAGATAAAGAA-3’), and *WWTR1* shRNA (sh*WWTR1*: 5’-GCGATGAATCAGCCTCTGAAT-3’) were designed and synthesized by OBIO Technology Company (Shanghai, China). For inducible expression, human *HSPA5* (NM_005347.4), *YAP1* (NM_006106), and *WWTR1* (NM_015472) cDNAs were cloned into the pSLenti-EF1-EGFP-F2A-Puro-CMV-MCS-WPRE vector (OBIO Technology Company, Shanghai, China). Full-length and deletion constructs of *HSPA5*, *YAP1*, and *WWTR1* were also subcloned into pcDNA3.1-3xFlag, pcDNA3.1-HA, or pcDNA3.1-Myc vectors (OBIO Technology Company, Shanghai, China). HA-tagged ubiquitin plasmids were sourced from Addgene. Small interfering RNAs (siRNAs) targeting β-TrCP were synthesized by GenePharma (Shanghai, China). The *β-TrCP* siRNA sequences were as follows: *β-TrCP* siNC: UUCUCCGAACGUGUCACGUTT; *β-TrCP* siRNA#1: GUGGAAUUUGUGGAACAUC. All constructs were verified by DNA sequencing, and transfections were performed using Lipofectamine^TM^ 3000 (Cat No. L3000150, ThermoFisher, MA, USA) according to the manufacturer’s protocols.

### RNA extraction and quantitative real‑time PCR

Total RNA was extracted using TRIzol^TM^ reagent (Cat No. 15596026CN, ThermoFisher, MA, USA) according to the manufacturer’s instructions. Briefly, cells were lysed in TRIzol, followed by chloroform extraction and isopropanol precipitation. The RNA pellet was washed with 75% ethanol, air-dried, and dissolved in RNase-free water. RNA purity and concentration were assessed using a NanoDrop 2000 spectrophotometer (ThermoFisher, USA). For cDNA synthesis, 1 µg of total RNA was reverse transcribed into cDNA using the HiScript Synthesis kit (#R212, Vazyme, Nanjing, China). Quantitative real-time PCR (qRT-PCR) was performed using SYBR® Green Master Mix (#Q321, Vazyme, Nanjing, China) on the StepOnePlus Real-Time PCR system (Applied Biosystems, USA) according to the preset amplification parameters. Relative gene expression levels were calculated using the 2^-ΔΔCt^ method, with *GAPDH* as an internal control. The primers used for qRT-PCR are listed in Table S4.

### Cell proliferation assay

Cell proliferation was assessed using the Cell Counting Kit-8 (Cat No. C0038, Beyotime, Shanghai, China). Briefly, cells were seeded into 96-well plates at a density of 1–2 × 10³ cells per well and cultured under standard conditions. At the indicated time points (24, 48, 72, and 96 h), 10 μL of CCK-8 reagent was added to each well and incubated for 1 h at 37 °C. Absorbance at 450 nm was measured using a microplate reader.

For the colony formation assay, cells were seeded into six-well plates (1 × 10^3^ cells/well) and cultured for 7–10 days until visible colonies appeared. Colonies were then fixed with 4% paraformaldehyde (#P1110, Solarbio, Beijing, China) for 30 min and stained with 0.2% crystal violet (#G1062, Solarbio, Beijing, China) for 30 min at room temperature. After washing and air drying, colonies containing more than 50 cells were manually counted or analyzed using ImageJ software.

### Transwell assay

To evaluate the effect of HSPA5 on the migration and invasion abilities of GBM cells, transwell assays were performed using Transwell chambers (Cat No. CLS3412, Corning, USA). A total of 5 × 10^4^ transfected GBM cells (200 μL) were seeded into the upper chamber that had been pre-coated with or without Matrigel (Cat No. 40183, Yeasen, Shanghai, China). The lower chamber was filled with 500 μL of complete medium containing 20% FBS. After 24 h of incubation, the cells on the lower surface of the chamber were fixed with 4% paraformaldehyde (#P1110, Solarbio, Beijing, China) and stained with 0.2% crystal violet (#G1062, Solarbio, Beijing, China). Cell migration and invasion were evaluated using a Leica microscope.

### Western blot

Cells and tissue were lysed in RIPA buffer (#P0013C, Beyotime, China) containing protease and phosphatase inhibitors (#P1045, Beyotime, China), and then incubated on ice for 30 min. Lysates were clarified by centrifugation at 12,000 × *g* for 15 min at 4 °C. Protein concentrations were quantified using the BCA Kit (#P0010S, Beyotime, China). Equal amounts of protein (20–40 μg) were separated on SDS-PAGE gels and transferred to polyvinylidene difluoride (PVDF) membranes (IPVH00010, Millipore). Membranes were blocked in 5% non-fat milk or BSA for 1 h at room temperature, followed by incubation with primary antibodies overnight at 4 °C. After washing, membranes were incubated with HRP-conjugated secondary antibodies at room temperature for 2 h. Protein bands were visualized using Super chemiluminescence (ECL) reagents (#S6009L, UElandy, China) and detected using the Chemiluminescent Imaging System (Tanon, China). The primary antibodies used for western blot analysis were as follows: HSPA5 (1:1000, Cat No. 66574-1-Ig, Proteintech, China), HSPA5 (1:1000, Cat No. 11587-1-AP, Proteintech, China), YAP (1:1000, Cat No. 13584-1-AP, Proteintech, China), TAZ (1:1000, Cat No. 23306-1-AP, Proteintech, China), p-YAP (S127) (1:1000, Cat No. 4911, Cell Signaling Technology, USA), p-TAZ (S89) (1:1000, Cat No. 59971, Cell Signaling Technology, USA), β-TrCP (1:1000, Cat No. 28393-1-AP, Proteintech, China), c-MET (1:1000, Cat No. 25869-1-AP, Proteintech, China), CD44 (1:1000, Cat No. 15675-1-AP, Proteintech, China), SOX2 (1:1000, Cat No. 11064-1-AP, Proteintech, China), OLIG2 (1:1000, Cat No. 13999-1-AP, Proteintech, China), Ub (1:2000, Cat No. A19686, Abclonal, China), GAPDH (1:5000, Cat No. 60004-1-Ig, Proteintech, China), GAPDH (1:5000, Cat No. 10494-1-AP, Proteintech, China), H3 (1:2000, Cat No. 17168-1-AP, Proteintech, China), GST (1:1000, Cat No. 10000-0-AP, Proteintech, China), Flag (1:1000, Cat No. 20543-1-AP, Proteintech, China), His (1:1000, Cat No. 66005-1-Ig, Proteintech, China), Myc (1:1000, Cat No. 60003-2-Ig, Proteintech, China).

### Nuclear and cytoplasmic protein isolation

Nuclear and cytoplasmic proteins were extracted using the Nuclear and Cytoplasmic Protein Extraction Kit (#P0027, Beyotime, China) according to the manufacturer’s instructions. In brief, harvested cells were washed three times with cold PBS and lysed in ice-cold cytoplasmic extraction buffer A supplemented with protease inhibitors. After 15 min incubation on ice, buffer B was added, and the sample was vortexed vigorously. The lysate was centrifuged at 12,000×*g* for 5 min at 4 °C to separate the cytoplasmic fraction (supernatant). The nuclear pellet was then resuspended in nuclear extraction buffer and vigorously vortexed every 2 min for a total of 30 min on ice, followed by centrifugation at 12,000×*g* for 10 min at 4 °C to collect the nuclear fraction (supernatant). Protein concentrations were determined using the BCA Kit (#P0010S, Beyotime, China). Finally, the proteins were separated by SDS-PAGE and subjected to western blot analysis.

### Co-immunoprecipitation (Co-IP)

Total cell lysates were prepared using RIPA lysis buffer (#P0013C, Beyotime, China) supplemented with protease inhibitors, followed by centrifugation at 12,000 × *g* for 10 min at 4 °C. The supernatants were incubated with 5 μg of the indicated primary antibody overnight at 4 °C with gentle rotation, along with 5 μg of mouse IgG (Cat No. B900620, Proteintech, China) or rabbit IgG antibodies (Cat No. 30000-0-AP, Proteintech, China) as negative controls. Next, 50 μL of pre-washed protein A/G magnetic beads (#36417ES03, Yeasen, China) were added and incubated for an additional 2–4 h. The magnetic beads were collected and washed three times with PBST. Then, the bound proteins were eluted by boiling in 1× SDS buffer for subsequent western blot analysis.

### GST pull-down assay

The GST, GST-HSPA5, GST-YAP, and GST-TAZ plasmids (OBIO Technology Company, Shanghai, China) were transfected into BL21 *E. coli* (#HY-15921, MCE, USA), and the culture was shaken overnight at 17 °C for protein amplification. The fusion protein was prepared and purified using glutathione agarose (#HY-K0211, MCE, USA) according to the manufacturer’s protocol. Then, the agarose was incubated with purified HSPA5, YAP, and TAZ proteins at 4 °C for 4-6 h on a rotating device. The bound proteins were analyzed by western blotting.

### Proximity ligation assay

The proximity ligation assay (PLA) was performed using a Duolink® In situ Detection Reagents Red kit (Sigma, Cat No. #DUO92101) following the manufacturer’s instructions. MES50 cells were seeded onto chamber slides, fixed with 4% paraformaldehyde, and permeabilized using 0.1% Triton X-100 in PBS. Non-specific binding sites were blocked with Duolink® Blocking Solution for 60 min at 37 °C in a humidified chamber. The cells were then incubated with primary antibodies against HSPA5, YAP, and TAZ overnight at 4 °C. Following primary antibody incubation, the cells were treated with Duolink® PLA probes for 1 h. Signal amplification was performed using the Duolink® amplification buffer for 100 min. The PLA signals were visualized under a Leica fluorescence microscope.

### Chromatin immunoprecipitation (ChIP)

Publicly available ChIP-seq data from the Cistrome database (http://cistrome.org/db/#/) were utilized to investigate potential TEAD1 binding sites in the promoter regions of CD44 and c-MET. ChIP assays were then performed using SimpleChIP® Plus Enzymatic Chromatin IP Kit (Cat No. #9004, Cell Signaling Technology, USA) according to the manufacturer’s instructions. Briefly, 1 × 10^7^ GBM cells were cross-linked with 1% formaldehyde for 10 min at room temperature, and the reaction was quenched with 0.125 M glycine (Cat No. #7005, Cell Signaling Technology, USA). Subsequently, lysis buffer was added to the cells to continue nuclear preparation and chromatin digestion. The processed chromatin was incubated with IgG or anti-TEAD1 (Cat No. A5218, Abclonal, China) antibodies at 4 °C overnight, followed by incubation with Protein A/G agarose for 2 h. The purified DNA samples were quantified via qRT-PCR analysis. The primer sequences used for ChIP-qPCR are provided in Table S5.

### Dual-luciferase reporter assays

HEK293T, MES50, or PN20 cells were co-transfected with 8xGTIIC-Luc or CTGF-Luc reporters and 200 ng of the indicated plasmids (shNC or sh*HSPA5*, Vector or *HSPA5*, and *HSPA5* wild-type or mutant) using Lipofectamine^TM^ 3000 (Cat No. L3000150, ThermoFisher, MA, USA). A Renilla luciferase plasmid (pRL, 5 ng) was also transfected as a transfection control. Forty-eight hours post-transfection, luciferase reporter expression was measured using the Dual-Luciferase Reporter Assay system (Cat No. #E1910, Promega, USA). The promoter activities were normalized to Renilla luciferase activity.

### Immunofluorescence assay

GBM cells were fixed with 4% paraformaldehyde (#P1110, Solarbio, Beijing, China) and permeabilized with 0.1% Triton X-100 (#T8200, Solarbio, Beijing, China) at room temperature for 15 min. Blocking was performed with 2.5% BSA solution (#A8020, Solarbio, Beijing, China). The cells were incubated with primary antibodies against YAP (1:200, Cat No. 13584-1-AP, Proteintech, China) and TAZ (1:200, Cat No. 23306-1-AP, Proteintech, China) overnight at 4 °C. After being washed three times, the cells were incubated with the corresponding secondary antibodies for 1 h at room temperature and stained with DAPI (1:1000, #62248, ThermoFisher, USA) for 5 min. Images were captured using a Leica microscope.

### Intracranial xenograft GBM models

Female BALB/c nude mice (4–6 weeks old, 18-20 g) were obtained from CrisBio Biotechnology Company (Nanjing, China) and randomly divided into experimental and control groups (eight mice per group). The mice were housed in a controlled environment with a 12-h light/12-h dark cycle, a temperature range of 20–26 °C, and relative humidity maintained at 30–70%. They had ad libitum access to food and water. Subsequently, 5 × 10^5^ stably transduced GBM cells were resuspended in 6 μL of precooled PBS and injected into the right cerebral hemisphere at 2 mm lateral and 1 mm posterior to the anterior fontanelle. Tumor growth was monitored using the IVIS Spectrum in vivo imaging system (PerkinElmer, USA) at the indicated times. Mice were humanely euthanized 4–7 weeks post-injection or upon reaching predefined ethical endpoints. Brains were harvested, fixed, and paraffin-embedded for hematoxylin and eosin (H&E) staining to confirm tumor presence and for subsequent immunohistochemical (IHC) analysis. For survival analysis, mice were monitored until manifestation of pathological symptoms caused by tumor burden developed (i.e., inactivity, reduced food consumption, loss of body weight, and hunched back) or 42 days after injection. All animal experiments were conducted according to the approved protocol by the Animal Welfare Ethical Review Committee of Affiliated Drum Tower Hospital of Nanjing University Medical School.

### Statistical analysis

All data were presented as means and standard deviations (SD) of at least three biological replicates. Unpaired or paired Student’s *t* test was used to determine the significance of differences. Correlations between the expression levels of *HSPA5*, *YAP1*, and *WWTR1* were assessed using Pearson correlation analysis. Statistical analysis was performed using GraphPad Prism software (version 9.0). A *P* value less than 0.05 was considered statistically significant.

## Results

### HSPA5 is highly expressed in GBM and is correlated with MES subtype

**Fig. 1:**
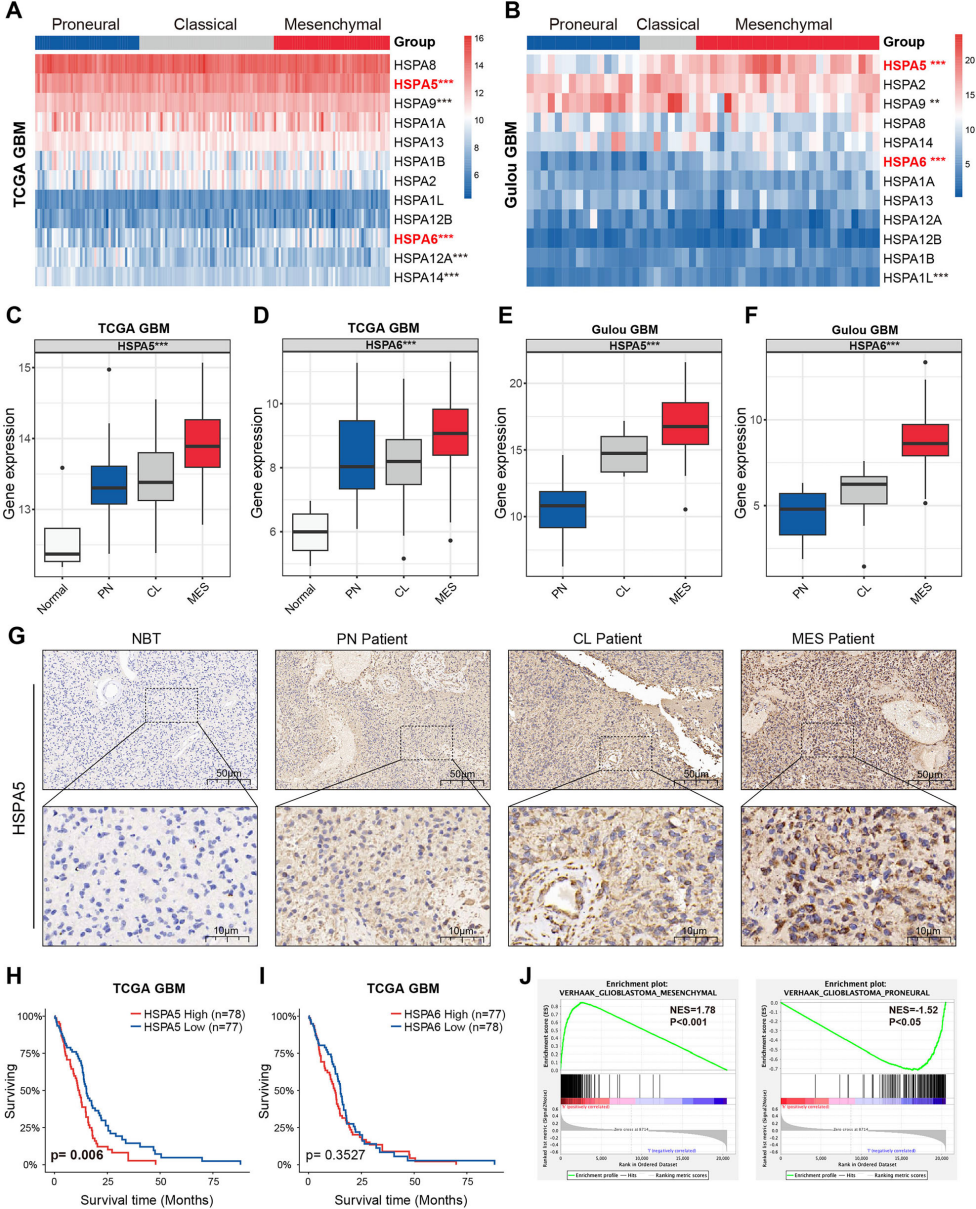
HSPA5 is highly expressed in GBM and is correlated with MES subtype. **A** Heatmap illustrating the mRNA expression levels of HSP70 family genes across Proneural (PN), Classical (CL), and Mesenchymal (MES) glioblastoma (GBM) subtypes from TCGA datasets. **B** Heatmap of HSP70 family gene expression in PN, CL, and MES subtypes from the Gulou GBM datasets. **C**, **D** The expression levels of *HSPA5* (**C**) and *HSPA6* (**D**) in normal brain tissues (NBTs) and GBM subtypes from TCGA dataset. **E**, **F** The expression levels of *HSPA5* (**E**) and *HSPA6* (**F**) in PN, CL, and MES GBM subtypes from the Gulou GBM datasets. **G** Representative immunohistochemical (IHC) staining images of HSPA5 protein in NBTs, PN, CL, and MES GBM samples. Scale bar: 50 μm. **H**, **I** Kaplan–Meier survival curves for GBM patients stratified by median *HSPA5* (**H**) and *HSPA6* (**I**) expression levels in TCGA cohort. P values were calculated using the log-rank test. **J** GSEA showed a significant positive correlation between the expression of *HSPA5* and MES subtypes, and a negative correlation with PN subtypes. ns: *P* > 0.05, *P < 0.05, **P < 0.01 and ***P < 0.001.

To identify potential HSP70 family proteins involved in PMT of GBM, we conducted differential expression analysis of TCGA and Gulou GBM datasets, which identified *HSPA5* and *HSPA6* as key candidates (Fig. 1A, B). Compared to normal tissue, HSPA5 and HSPA6 expression levels were elevated in GBM tissues, especially in MES subtype (Figs. 1C–F and S1A, B). Immunohistochemistry (IHC) analysis further confirmed that HSPA5 expression was highest in MES subtype (Figs. 1G and S1C). Moreover, high *HSPA5* expression was significantly associated with poor prognosis, whereas *HSPA6* expression was not associated with prognosis (Fig. 1H, I). Therefore, we focused on *HSPA5* for further functional investigations. Gene Set Enrichment Analysis (GSEA) revealed that *HSPA5* expression was strongly associated with GLIOBLASTOMA_MESENCHYMAL pathway, and negatively correlated with GLIOBLASTOMA_PRONEURAL pathway (Fig. 1J). These results demonstrated that HSPA5 was highly expressed in GBM and was correlated with aggressive MES subtypes.

### HSPA5 may be involved in regulating PMT

**Fig. 2:**
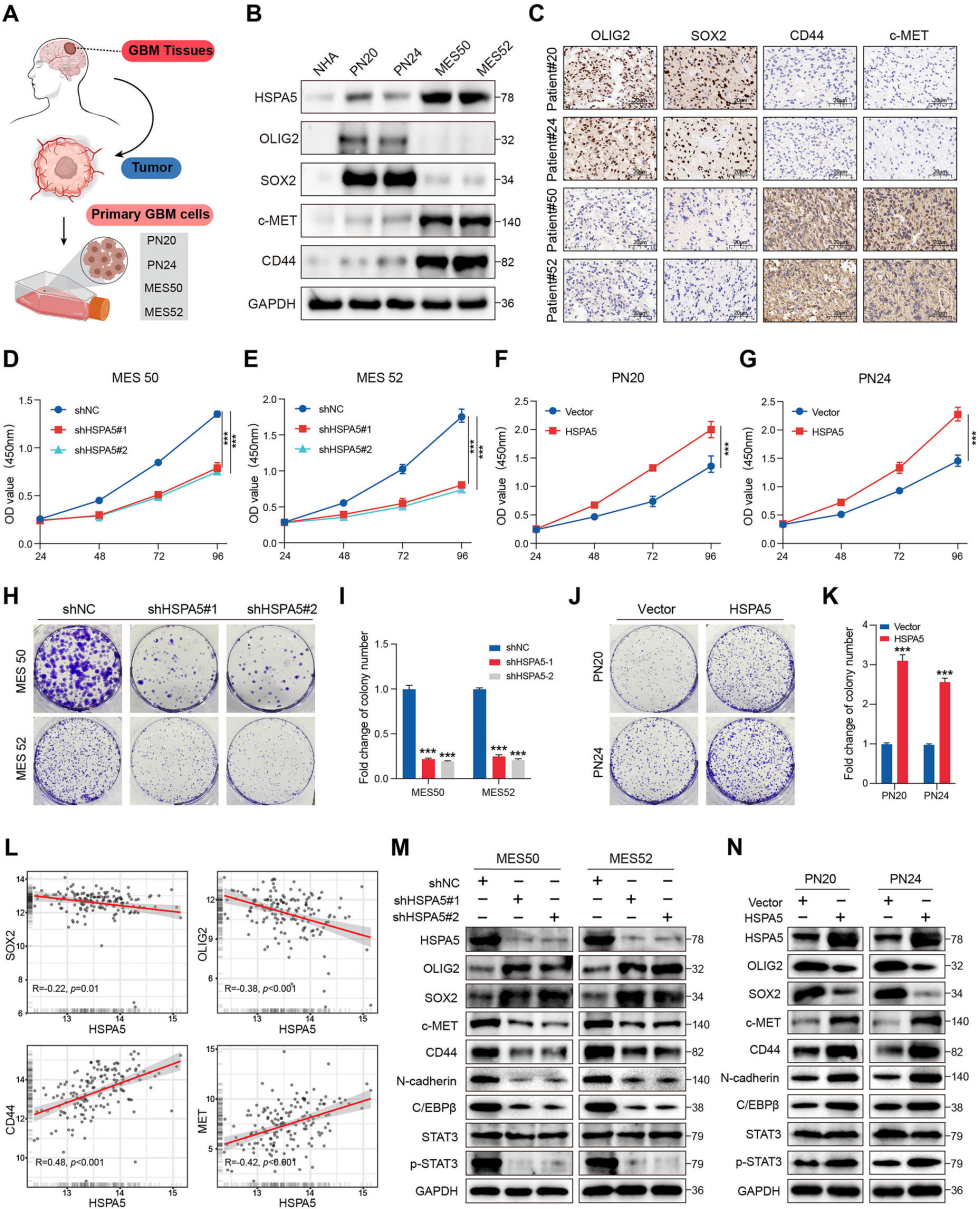
HSPA5 may be involved in regulating PMT. **A** Primary GBM cells (PN20, PN24, MES50 and MES52) were isolated from post-surgical GBM tissues. **B** Western blot (WB) analysis of HSPA5, OLIG2, SOX2, c-MET, and CD44 protein expression in NHA, PN (PN20 and PN24), and MES (MES50 and MES52) cells. **C** Representative IHC staining of OLIG2, SOX2, CD44, and c-MET in clinical PN and MES GBM specimens. Scale bar: 20 μm. **D**, **E** CCK-8 assay was used to analyze the cell viability of MES cells transduced with shNC and shHSPA5. **F**, **G** CCK-8 assay was used to analyze the cell viability of PN cells transduced with Vector and HSPA5. **H**, **I** Colony formation assay was used to analyze the cell proliferation ability of MES cells transduced with shNC and shHSPA5. **J**, **K** Colony formation assay was used to analyze the cell proliferation ability of PN cells transduced with Vector and HSPA5. **L** Spearman correlation analysis between *HSPA5* expression and PN (*OLIG2* and *SOX2*) or MES (*CD44* and *c-MET*) subtype biomarkers in TCGA GBM datasets. **M** WB analysis of HSPA5, OLIG2, SOX2, c-MET, CD44, C/EBPβ, N-cadherin, STAT3, and p-STAT3 protein levels in MES cells following HSPA5 knockdown. **N** WB analysis of HSPA5, OLIG2, SOX2, c-MET, CD44, C/EBPβ, N-cadherin, STAT3, and p-STAT3 protein levels in PN cells following HSPA5 overexpression. Data are shown as mean ± SD. *P < 0.05, **P < 0.01 and ***P < 0.001. Two-tailed unpaired *t* test (**D**–**K**).

To elucidate the function of *HSPA5*, we isolated primary GBM cells (PN20, PN24, MES50, and MES52) from post-surgical GBM tissues for subsequent in vivo or in vitro experiments (Fig. 2A). Notable morphological differences were observed between PN and MES cells (Fig. S2A). IHC and WB results revealed that the MES subtype exhibited higher levels of CD44 and c-MET, whereas PN subtype predominantly expressed OLIG2 and SOX2 proteins, consistent with previous studies [7] (Fig. 2B, C). Furthermore, HSPA5 expression was significantly higher in MES50 and MES52 cells than in PN20 and PN24 cells (Fig. S2B). CCK8 (Fig. 2D–G) and colony formation assays (Fig. 2H–K) showed that HSPA5 regulates cell proliferation (Fig. S2C, D), as well as cell migratory and invasive capacities of GBM cells (Fig. S2E–L). Notably. HSPA5 expression was positively correlated with MES markers and negatively associated with PN markers in TCGA datasets (Fig. 2L). Western blot analysis demonstrated that HSPA5 knockdown markedly reduced CD44, c-MET, and p-STAT3 protein levels in MES cells, while overexpression of HSPA5 in PN cells yielded the opposite effect (Fig. 2M, N). Taken together, we speculated that HSPA5 promotes malignant phenotypes by regulating PMT in GBM cells.

### HSPA5 facilitates PMT in GBM by modulating YAP/TAZ

**Fig. 3:**
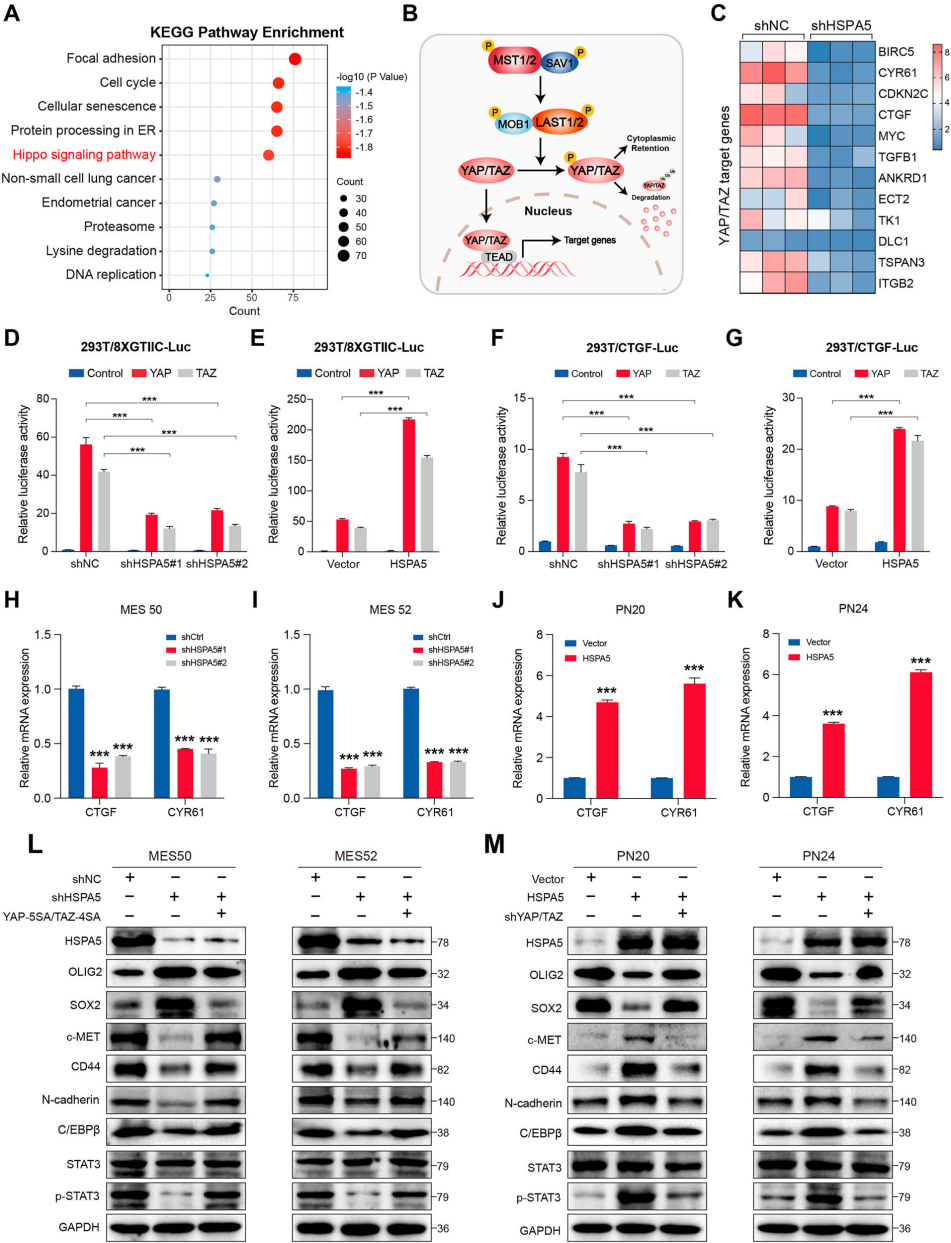
HSPA5 facilitates PMT in GBM by modulating YAP/TAZ. **A** The RNA-seq results were used to identify the enriched pathways after knockdown of *HSPA5* expression in MES50 cells. **B** A schematic illustration of the Hippo pathway in mammals. The activated LATS1/2 phosphorylated and inactivated YAP/TAZ, preventing it from translocating into the nucleus and binding to transcription factors TEAD1–4. **C** Heatmap showing expression levels of YAP/TAZ downstream target genes in MES50-shNC and MES50-shHSPA5 cells. **D** Effect of *HSPA5* knockdown (shNC, shHSPA5#1, and shHSPA5#2) on 8xGTIIC-Luc activity in HEK293T cells transfected with YAP or TAZ expression vectors. **E** Effect of *HSPA5* overexpression (Vector and HSPA5) on 8xGTIIC-Luc activity in HEK293T cells transfected with YAP or TAZ expression vectors. **F** Effect of *HSPA5* knockdown (shNC, shHSPA5#1, and shHSPA5#2) on CTGF Luc reporter transcriptional activity in HEK293T cells transfected with YAP or TAZ expression vectors. **G** Effect of *HSPA5* overexpression (Vector and HSPA5) on CTGF Luc reporter transcriptional activity in HEK293T cells transfected with YAP or TAZ expression vectors. **H**, **I** qRT-PCR analysis of the expression of YAP/TAZ downstream target genes (*CTGF* and *CYR61*) in MES cells transduced with shNC and shHSPA5. **J**, **K** qRT-PCR analysis of the expression of YAP/TAZ downstream target genes (*CTGF* and *CYR61*) in PN cells transduced with Vector and HSPA5. **L** WB analysis of protein expression of HSPA5, OLIG2, SOX2, c-MET, CD44, C/EBPβ, N-cadherin, STAT3, and p-STAT3 in shNC/shHSPA5 MES cells treated with indicated interventions (transfection of unphosphorylatable YAP-5SA/TAZ-4SA). **M** WB analysis of protein expression of HSPA5, OLIG2, SOX2, c-MET, CD44, C/EBPβ, N-cadherin, STAT3, and p-STAT3 in Vector/HSPA5 PN cells treated with indicated interventions (knockdown of YAP/TAZ). Data are shown as mean ± SD. *P < 0.05, **P < 0.01 and ***P < 0.001. Two-tailed unpaired *t* test (**D**–**K**).

To investigate the mechanism underlying HSPA5-induced PMT, we conducted RNA-seq analysis on MES50-shNC and MES50-sh*HSPA5* cells. GSEA and single-sample gene set enrichment analysis (ssGSEA) of the RNA-seq data indicated that HSPA5 mediates PMT in GBM cells at the transcriptome level (Fig. S3A, B). KEGG pathway enrichment analysis revealed that *HSPA5* depletion affected the Hippo signaling pathway (Fig. 3A). YAP and TAZ, the key downstream effectors of the Hippo pathway, serve as crucial regulators of PMT in GBM [29–32] (Fig. 3B). The heatmap showed that *HSPA5* deficiency substantially reduced mRNA expression of multiple YAP/TAZ target genes (Fig. 3C). Notably, luciferase reporter assays indicated that *HSPA5* knockdown inhibited YAP/TAZ transcriptional activity, whereas ectopic expression of *HSPA5* enhanced this activity (Fig. 3D–G). *HSPA5* knockdown significantly decreased expression of YAP/TAZ target genes in MES cells (Fig. 3H, I), while *HSPA5*-overexpressing PN cells exhibited elevated expression of these genes (Fig. 3J, K). Additionally, depletion of YAP/TAZ did not affect HSPA5 mRNA and protein levels (Fig. S3C). These results indicated that HSPA5-induced PMT may be a consequence of YAP/TAZ activation. To confirm this hypothesis, we carried out a series of rescue experiments. CCK8, colony formation, and Transwell assays demonstrated that *HSPA5* knockdown inhibited MES cell proliferation, migration, and invasion, and these effects were rescued by overexpression of YAP-5SA/TAZ-4SA (unphosphorylatable YAP/TAZ mutants) (Fig. S3D–G). Conversely, YAP/TAZ depletion reversed HSPA5-induced proliferation, migration, and invasion in PN cells (Fig. S3H–K). Depletion of HSPA5 led to decreased expression of MES markers (CD44 and c-MET) and increased PN markers (OLIG2 and SOX2), which were reversed by ectopic expression of YAP-5SA/TAZ-4SA (Fig. 3L). As expected, downregulation of YAP/TAZ significantly attenuated HSPA5-induced PMT (Fig. 3M). Furthermore, YAP/TAZ depletion reduced both the mRNA and protein expression of c-MET and CD44 in MES cells (Fig. S4A–C), suggesting that these MES markers may be transcriptionally regulated by YAP/TAZ.

Given that TAZ lacks a DNA-binding domain, it functions as a transcriptional coactivator through interaction with TEAD transcription factors (TEAD1-4), ultimately driving YAP/TAZ-TEAD-mediated transcription [21]. By analyzing all TEAD family members in TCGA GBM and Gulou GBM datasets, we observed that *TEAD1* was the most abundantly expressed member (Fig. S4D, E). To further investigate, we analyzed publicly available ChIP-seq data from the Cistrome database (http://cistrome.org/db/#/), which revealed that *TEAD1* binds at the promoter regions of *CD44* and *c-MET* (Fig. S4F, G). JASPAR (https://jaspar.elixir.no/) database analysis identified two conserved TEAD1-binding motifs in each promoter (Fig. S4H). ChIP-qPCR confirmed that YAP/TAZ bind to specific regions of the CD44 (Region 1) and c-MET promoter (Region 1) in MES cells (Fig. S4I, J). Additionally, dual-luciferase reporter assays indicated that YAP/TAZ overexpression enhances CD44 and c-MET promoter activity, while mutation of their respective Region 1 nearly attenuated this activation (Fig. S4K, L). These results confirmed that CD44 and c-MET were regulated by YAP/TAZ at the transcriptional level. Collectively, these findings demonstrated that HSPA5 promotes PMT by activating YAP/TAZ.

### HSPA5 stabilizes YAP/TAZ independent of the Hippo pathway

**Fig. 4:**
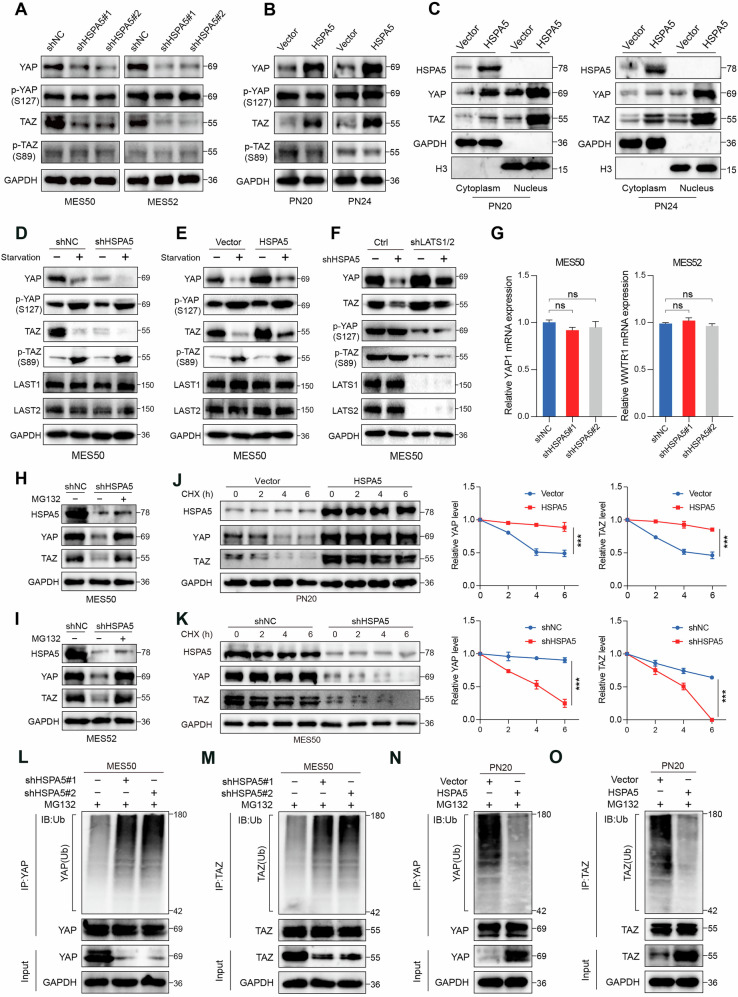
HSPA5 stabilizes YAP/TAZ independent of the Hippo pathway. **A**, **B** WB analysis of total YAP, TAZ, and their phosphorylated forms (p-YAP S127, and p-TAZ S89) protein levels in MES (A) and PN (B) cells following HSPA5 knockdown or overexpression. **C** WB analysis of YAP and TAZ protein levels in cytoplasmic and nuclear fractions from indicated cells. GAPDH and Histone H3 serve as fractionation controls. **D**, **E** WB analysis of the Hippo pathway components in serum-starved MES (**D**) and PN (**E**) cells following HSPA5 knockdown or overexpression. **F** WB analysis of YAP/TAZ in control (shNC) and LATS1/2 double-knockdown (shLATS1/2) MES50 cells with or without HSPA5 knockdown. **G** qRT-PCR analysis of *YAP1* and *WWTR1* mRNA levels in MES cells transduced with sh*NC* and sh*HSPA5* knockdown. **H**, **I** WB analysis of YAP/TAZ protein levels in HSPA5-knockdown MES cells treated with proteasome inhibitor MG132 (20 μM) or DMSO vehicle control for 8 h. **J**, **K** WB analysis of total lysates derived from PN20 and MES50 cells stably expressing empty vector, HSPA5, shNC or shHSPA5 and treated with cycloheximide (CHX, 20 μg/mL) for the indicated time points. GAPDH was analyzed as an internal loading control. Quantification of the band intensities normalized to the *t* = 0 controls is shown in the right panel. **L**, **M** WB analysis of total lysates and immunoprecipitates from HSPA5 knockdown MES50 cells. Cells were treated with MG132 (20 µM for 8 h), and the endogenous poly-ubiquitinated YAP (**L**) and TAZ (**M**) were immunoprecipitated with anti-YAP or anti-TAZ antibodies, respectively, and immunoblotted with anti-Ub antibodies. **N**, **O** WB analysis of total lysates and immunoprecipitates from HSPA5 overexpression PN20 cells. Cells were treated with MG132 (20 µM for 8 h), and the endogenous poly-ubiquitinated YAP (**N**) and TAZ (**O**) were immunoprecipitated with anti-YAP or anti-TAZ antibodies, respectively, and immunoblotted with anti-Ub antibodies. Data are shown as mean ± SD. *P < 0.05, **P < 0.01 and ***P < 0.001. Two-tailed unpaired *t* test (**G**, **J**, and **K**).

To explore the molecular mechanism by which HSPA5 regulates YAP/TAZ signaling, we examined whether HSPA5 modulates YAP/TAZ protein expression. Intriguingly, HSPA5 knockdown significantly reduced YAP/TAZ protein levels in MES cells (Fig. 4A), whereas HSPA5 overexpression elevated YAP/TAZ levels in PN cells without affecting phosphorylated YAP (S127) or TAZ (S89) (Fig. 4B). This regulatory effect of HSPA5 on YAP/TAZ protein expression was consistently observed across multiple cancer cell lines (Fig. S5A–F). Furthermore, HSPA5 overexpression increased the nuclear accumulation of YAP/TAZ (Figs. 4C and S5G), as confirmed by immunofluorescence analysis (Fig. S5H). Previous research has indicated that YAP/TAZ upregulation can occur through either Hippo pathway dysregulation or a post-transcriptional modification [33, 34]. Given that LATS1/2 kinases are classical regulators of the Hippo pathway, and that LATS-dependent phosphorylation promotes YAP/TAZ cytoplasmic localization, ubiquitination, and degradation [35]. Next, we investigated whether the regulatory effect of HSPA5 on YAP/TAZ is associated with the previously described control of YAP/TAZ by the Hippo pathway. Serum deprivation–induced activation of the Hippo pathway markedly decreased YAP and TAZ protein abundance in GBM cells, with a more pronounced reduction observed in HSPA5-deficient cells (Fig. 4D). However, overexpression of HSPA5 effectively prevented the serum starvation–induced degradation of YAP and TAZ without altering their phosphorylation status (Fig. 4E). These results indicated that HSPA5 likely controls YAP/TAZ independently of MST and LATS. To determine whether HSPA5 directly regulates YAP/TAZ protein levels through their upstream kinases MST1/2 or LATS1/2, we compared shCtrl and shMST1/2 or shLATS1/2-double knockdown MES50 cells. Interestingly, HSPA5 knockdown continued to diminish YAP and TAZ expression even after MST1/2 or LATS1/2 depletion (Figs. 4F and S5I), indicating that HSPA5 regulates YAP/TAZ stabilization independent of the canonical Hippo kinase cascade.

As a member of the HSP70 family, HSPA5 is known to modulate protein stability [36]. We found that *YAP1* and *WWTR1* mRNA levels were not affected by *HSPA5* knockdown (Fig. 4G), suggesting that HSPA5 may maintain YAP/TAZ protein stability via post-transcriptional modification. To verify the above speculation, we found that the proteasome inhibitor MG132 (20 μM) partially restored the reduction in YAP/TAZ protein levels induced by HSPA5 knockdown (Fig. 4H, I). Cycloheximide pulse-chase assay showed that HSPA5 overexpression prolonged the half-life of endogenous YAP/TAZ (Fig. 4J), while HSPA5 knockdown accelerated the degradation of YAP/TAZ and shortened its half-life (Fig. 4K). Importantly, depletion of HSPA5 enhanced the ubiquitination of endogenous YAP/TAZ (Fig. 4L, M), whereas HSPA5 overexpression reversed these effects (Fig. 4N, O). Collectively, these results suggested that HSPA5 stabilizes YAP/TAZ by inhibiting ubiquitin proteasomal mediated degradation.

### HSPA5 SBD domain stabilizes YAP/TAZ by disrupting its interaction with β-TrCP

**Fig. 5:**
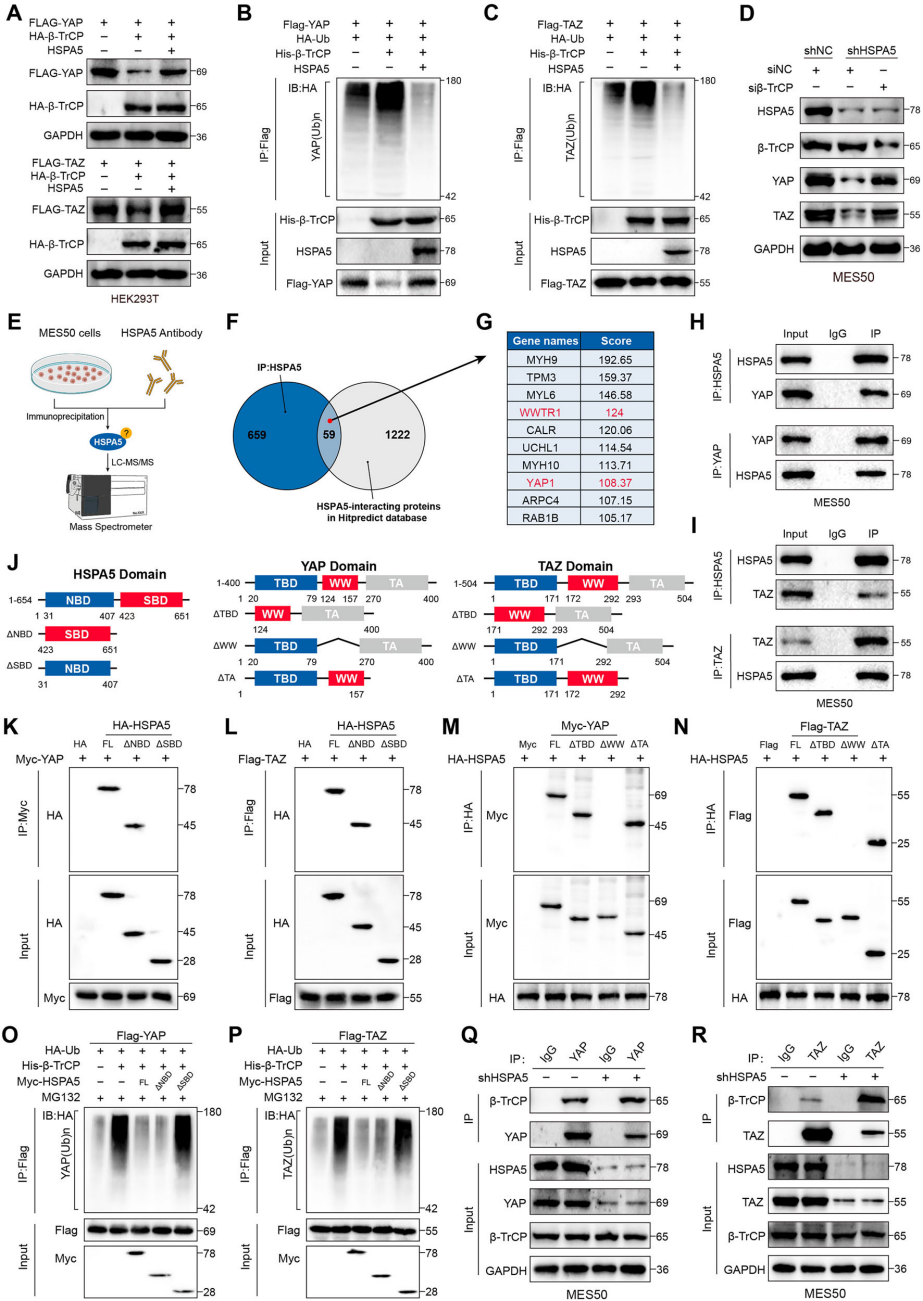
HSPA5 SBD domain stabilizes YAP/TAZ by disrupting its interaction with β-TrCP. **A** WB analysis of total lysates from HEK293T cells co-transfected with FLAG-YAP or FLAG-TAZ, HA-β-TrCP, and HSPA5 expression vectors as indicated. Cells were treated with MG132 (20 µM) for 8 h prior to lysis. **B**, **C** WB analysis of total lysates and immunoprecipitates from HEK293T cells stably expressing FLAG-YAP (**B**) or FLAG-TAZ (C), HA-Ub, HA-β-TrCP, and HSPA5. Cells were treated with MG132 (20 µM for 8 h), Flag-tagged proteins were immunoprecipitated, and poly-ubiquitination was detected using anti-HA-Ub antibodies. **D** WB analysis of protein expression of HSPA5, β-TrCP, YAP and TAZ in HSPA5-depletion MES50 cells and transfected with siNC and siβ-TrCP siRNA. **E** Schematic workflow for the identification of HSPA5-interacting proteins. Endogenous HSPA5 was immunoprecipitated from MES50 cell lysates, and bound proteins were identified by liquid chromatography-tandem mass spectrometry (LC-MS/MS). **F** The Venn diagram showing the overlap (n = 59) between HSPA5-interacting proteins identified by mass spectrometry and predicted interactors from the HitPredict database. **G** The table presents the rankings and predicted scores of the top 10 peptides, including peptides derived from YAP and TAZ proteins. **H**, **I** Endogenous co-immunoprecipitation (Co-IP) in MES50 cells. Cell lysates were immunoprecipitated with anti-YAP (**H**), anti-TAZ (**I**), or anti-HSPA5 (**I**) antibodies, and corresponding IgG controls. Immunoprecipitates and inputs were analyzed by WB. **J** Schematic representation of the domain structures of full-length and deletion mutants of HSPA5, YAP, and TAZ used in this study. **K**, **L** Plasmids containing full-length and truncated HSPA5 constructs were generated, and HEK293T cells were transfected with the indicated plasmids. The Co-IP assay was performed to explore the binding regions between HSPA5 and YAP (**K**) or TAZ (**L**). **M**, **N** Plasmids containing full-length and truncated YAP and TAZ constructs were generated, and HEK293T cells were transfected with the indicated plasmids. The Co-IP assay was performed to explore the binding regions between HSPA5 and YAP (**M**) or TAZ (**N**). **O**, **P** The ubiquitination assay was conducted using exogenously expressed HA-Ub, His-β-TrCP, FLAG-YAP or FLAG-TAZ, and the indicated HSPA5 truncation mutants. Cells were treated with MG132 (20 µM for 8 h), and immunoprecipitation was performed to assess the ubiquitination of TAZ (**O**) and TAZ (**P**). **Q**, **R** Endogenous Co-IP assays were performed in MES50-shNC and MES50-shHSPA5 cells following MG132 (20 µM for 8 h) treatment. The interaction between β-TrCP and YAP (**Q**) or TAZ (**R**) were subsequently examined.

Previous studies have shown that β-TrCP is a well-established E3 ubiquitin ligase that mediates the ubiquitination and degradation of YAP/TAZ [7, 37]. Notably, HSPA5 overexpression effectively attenuated both the β-TrCP-induced ubiquitination and the suppression of YAP/TAZ transcriptional activity (Figs. 5A–C and S6A). Consistent with this observation, β-TrCP knockdown (Fig. S6B) rescued YAP/TAZ protein levels and transcriptional activity in HSPA5-deficient MES cells (Figs. 5D and S6C, D). Moreover, knockdown of HSPA5 reduced both the protein and mRNA levels of CD44 and c-MET, which were reversed by β-TrCP silencing (Fig. S6E–G). Functionally, the impairment in cell proliferation caused by HSPA5 depletion was rescued by β-TrCP knockdown (Fig. S6H, I). Similarly, Transwell assay demonstrated that β-TrCP silencing partially restored migration and invasion abilities in HSPA5-depleted MES cells (Fig. S6J, K).

Proteins with specific functions can bind and prevent YAP/TAZ degradation induced by β-TrCP [34, 37]. Therefore, we speculated that HSPA5 might prevent β-TrCP-induced YAP/TAZ degradation via binding to YAP/TAZ. To identify potential interactors, we performed mass spectrometry and integrated the results with the HitPredict database (https://www.hitpredict.org/index.html), which revealed 59 HSPA5-interacting proteins, including YAP and TAZ (Fig. 5E–G) (Tables S6 and 7). Endogenous co-immunoprecipitation (Co-IP) and GST pull-down assays confirmed that HSPA5 strongly interacts with YAP and TAZ (Figs. 5H, I and S7A–D). The duolink-PLA assay was performed to determine whether HSPA5 could interact with YAP/TAZ in GBM cells. The results showed significantly increased red dots caused by the interaction between HSPA5 and YAP/TAZ (Fig. S7E, F). Moreover, the interaction was enhanced upon inactivation of the Hippo pathway via LATS1/2 knockdown (Fig. S8A). Importantly, molecular docking indicated that the SBD domain of HSPA5 may bind to the WW domain of YAP and TAZ (Fig. S8B). We next generated domain deletion mutants of HSPA5, YAP, and TAZ proteins (Fig. 5J). Co-IP assay indicated the HSPA5 FL and HSPA5 ΔNBD deletion mutants retained their binding capability to YAP and TAZ, whereas the HSPA5 ΔSBD mutant lost this ability, indicating that the SBD domain of HSPA5 was necessary for binding to YAP and TAZ. (Fig. 5K, L). For YAP and TAZ, the WW domain was essential for their interaction with HSPA5 (Fig. 5M, N). Overexpression of HSPA5 FL and NBD domain deletion mutant decreased β-TrCP-mediated ubiquitination of YAP and TAZ, while deletion of the SBD domain eliminated this effect (Fig. 5O, P). Consistently, we found that HSPA5 ΔSBD impaired protein levels and transcriptional activity of YAP/TAZ (Fig. S8C–E). Additionally, overexpression of the SBD domain deletion mutant abolished the cell proliferation, migration, and invasion ability induced by HSPA5 full-length and NBD domain deletion mutant (Fig. S8F–H). To determine whether HSPA5 binds to YAP/TAZ and prevents its interaction with β-TrCP, we found that knockdown of HSPA5 increased the interaction between β-TrCP and YAP/TAZ (Fig. 5Q, R), while HSPA5 overexpression disrupted this interaction in a dose-dependent manner (Fig. S8I, J). In summary, these results suggested that the HSPA5 SBD domain interacts with and stabilizes YAP/TAZ by disrupting their interaction with β-TrCP, thereby preventing their ubiquitination and proteasomal degradation.

### HSPA5 facilitates PMT in GBM by modulating YAP/TAZ in vivo

**Fig. 6:**
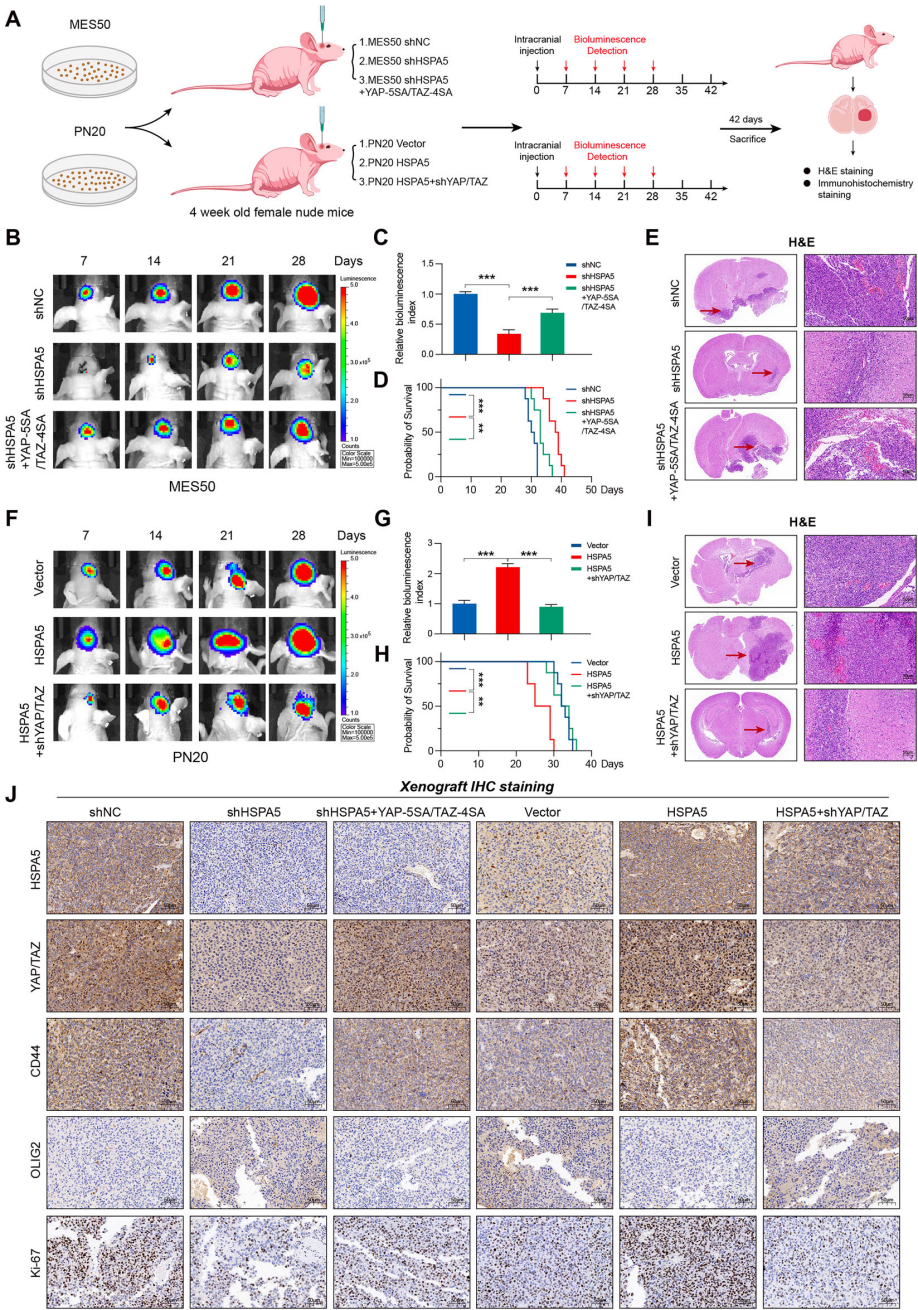
HSPA5 facilitates PMT in GBM by modulating YAP/TAZ in vivo. **A** A schematic of the experimental procedure for establishing the GBM intracranial xenograft mouse model. **B**–**D** Bioluminescence images (**B**), quantification of bioluminescence intensity at day 28 (**C**), and Kaplan–Meier survival curves (**D**) of mice implanted with luciferase-expressing MES50 cells expressing shNC, shHSPA5, or shHSPA5 + YAP-5SA/TAZ-4SA. *n* = 8 mice per group. **E** Representative H&E-staining images of nice intracranial GBM xenograft models derived from luciferase-expressing MES50 cells transfected with indicated vectors. Scale bar: 50 μm. **F**–**H** Bioluminescence images (**F**), quantification bioluminescence intensity at day 28 (**G**), and Kaplan–Meier survival curves (**H**) of mice implanted with luciferase-expressing PN20 cells expressing Vector, HSPA5, and HSPA5+shYAP/TAZ. *n* = 8 mice per group. **I** Representative H&E-staining images of nice intracranial GBM xenograft models derived from luciferase-expressing PN20 cells transfected with indicated vectors. Scale bar: 50 μm. **J** Immunohistochemical staining was used to examine the expression levels of HSPA5, YAP/TAZ, CD44, OLIG2, and Ki-67 in xenograft tumor tissues from different experimental groups. Scale bar: 50 μm. Data are shown as mean ± SD. *P < 0.05, **P < 0.01 and ***P < 0.001. Two-tailed unpaired *t* test (**C**, **G**). Log-rank test (**D**, **H**).

To investigate whether HSPA5 induces the PMT in vivo, we established a GBM xenograft model using nude mice. A schematic diagram illustrating the study flow of the animal experiment is shown in Fig. 6A. Luciferase-labeled MES50 and PN20 GBM cells were transfected with lentiviral vectors and injected into the brains of mice. Tumor growth was monitored using an IVIS system at 7, 14, 21, and 28 days. Bioluminescence images indicated that *HSPA5* knockdown inhibited tumor growth, and this effect was reversed by ectopic expression of YAP-5SA/TAZ-4SA (Fig. 6B, C, E). Kaplan–Meier survival analysis revealed that YAP-5SA/TAZ-4SA expression counteracted the shortened survival induced by HSPA5 knockdown (Fig. 6D). Conversely, YAP/TAZ depletion in HSPA5-overexpressing GBM xenografts exhibited the inverse effects (Fig. 6F–I). In addition, immunohistochemical staining of xenograft tumor tissues showed expression patterns of HSPA5, YAP, TAZ, CD44, N-cadherin, CEBPβ, and OLIG2 consistent with previous experiments (Figs. 6J and S8K). Together, these findings indicated that HSPA5 promotes PMT by activating YAP/TAZ in vivo.

### Clinical relevance of HSPA5-YAP/TAZ driven PMT in human GBM

**Fig. 7:**
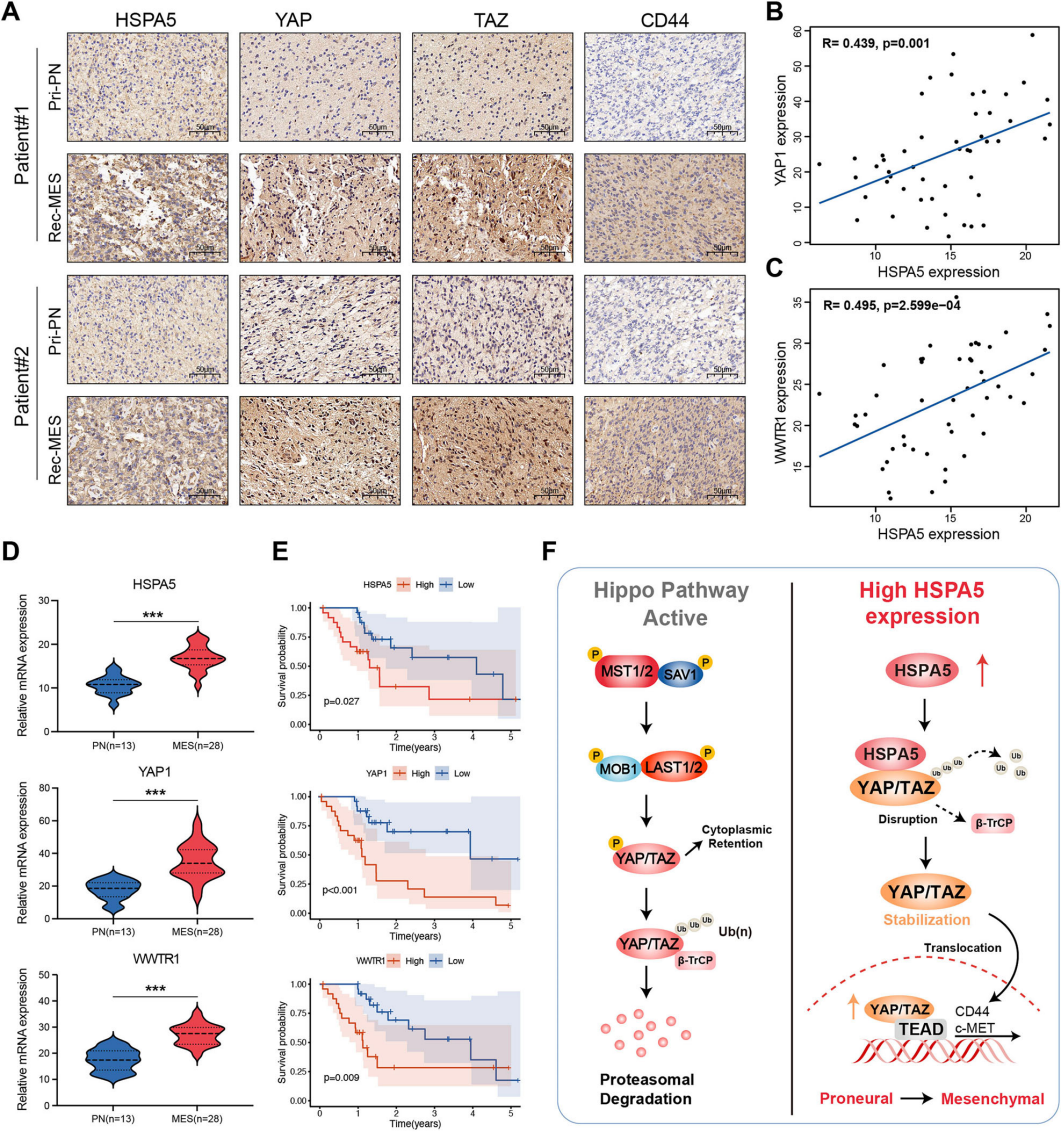
Clinical relevance HSPA5-YAP/TAZ driven PMT in human GBM. **A** Representative image showing HSPA5, YAP, and TAZ protein expression in two matched pairs of primary (PN subtype) and their corresponding recurrent (MES subtype) GBM specimens. Scale bar: 50 μm. **B**, **C** Spearman correlation analysis between *HSPA5* and *YAP1* or *WWTR1* mRNA levels in the Gulou GBM datasets (*n* = 50 patient samples). **D** qRT-PCR analysis of *HSPA5*, *YAP1*, and *WWTR1* mRNA levels in PN and MES GBM specimens from the Gulou cohort. **E** Kaplan–Meier survival analysis for GBM patients in the Gulou cohort stratified by median *HSPA5*, *YAP1*, and *WWTR1* expression levels in GBM. *P* values were calculated using the log-rank test. **F** Schematic illustration of the current study: HSPA5 interacts with and stabilizes YAP/TAZ by disrupting their interaction with β-TrCP, thereby promoting PMT in GBM. Data are shown as mean ± SD. *P < 0.05, **P < 0.01 and ***P < 0.001. Two-tailed unpaired *t* test (**D**–**F**).

To further evaluate the clinical significance of the HSPA5-YAP/TAZ axis, we analyzed the expression levels of HSPA5, YAP, TAZ, and CD44 in primary PN subtype GBM specimens and their corresponding recurrent MES subtype specimens. As expected, HSPA5, YAP, TAZ, and CD44 were highly expressed in recurrent MES specimens (Fig. 7A). Spearman’s correlation analysis revealed a positive correlation between *HSPA5* and *YAP1/WWTR1* expression levels in GBM samples (Fig. 7B, C). Additionally, compared to PN samples, the mRNA levels of *HSPA5*, *YAP1*, and *WWTR1* were significantly higher in MES samples (Fig. 7D–F). Survival analysis demonstrated that the high expression of *HSPA5*, *YAP1*, and *WWTR1* was associated with poorer prognosis (Fig. 7G–I). Taken together, these results supported our experimental findings that HSPA5 stabilizes YAP/TAZ protein and promotes PMT in GBM.

## Discussion

Glioblastoma is classified into three transcriptional subtypes: proneural (PN), classical (CL), and mesenchymal (MES) [38]. The transition from the PN to the MES subtype can occur in patients after undergoing chemotherapy and radiotherapy. Notably, the molecular events driving this shift are similar to those driving EMT in tumor cells [1], and this transition is associated with heightened tumor aggressiveness and increased recurrence rates [39, 40]. While some regulatory factors have been reported to modulate PMT, the precise molecular mechanisms underlying this process remain unclear.

In this study, we investigated the role of HSPA5 in primary GBM cells. Our results demonstrated that HSPA5 functions as a regulator to promote PMT and maintain MES characteristics in GBM. We found that HSPA5 was highly expressed in MES subtype of GBM. Knockdown of HSPA5 in MES cells leads to the loss of MES features and inhibits malignant behavior, whereas overexpression of HSPA5 results in acquisition of MES characteristics in PN cells. Mechanistically, we discovered that HSPA5 promotes the stabilization and transcriptional activity of YAP/TAZ by inhibiting their proteasomal degradation. HSPA5 stabilizes YAP/TAZ proteins by disrupting their interaction with β-TrCP. Rescue experiments confirmed that HSPA5 promotes PMT by upregulating YAP/TAZ levels both in vitro and in vivo.

*HSPA5*, a member of the HSP70 family, functions as an endoplasmic reticulum chaperone protein, regulating unfolded protein responses to maintain endoplasmic reticulum homeostasis [13, 41]. Previous research has shown that HSPA5 plays a crucial role in the oncogenic process of various cancers [17, 36]. Consistent with these studies, we found that overexpression of HSPA5 enhances the proliferation and migratory ability of GBM cells. PMT is driven by the activation of master regulators, including STAT3, NF-κB, C/EBPβ, and TAZ [40, 42]. Numerous studies indicated that YAP/TAZ are involved in modulating PMT and are positively correlated with the malignant subtype of GBM [28, 43–45]. Our study revealed that overexpression of HSPA5 in PN cells leads to the stabilization and increased transcriptional activity of YAP/TAZ, promoting the acquisition of MES characteristics. Additionally, the MES markers, CD44 and c-MET were transcriptionally regulated by YAP/TAZ. These results demonstrated that HSPA5 induces PMT by modulating YAP/TAZ.

Furthermore, we considered the potential crosstalk between the HSPA5-YAP/TAZ axis and other key drivers of PMT. These core regulators rarely operate in isolation but often form synergistic or positive feedback loops that collectively maintain the MES phenotype. For instance, extensive crosstalk exists between YAP/TAZ and the STAT3 signaling pathway, which YAP/TAZ can facilitate STAT3 activation or cooperate with STAT3 to regulate a shared set of pro-tumorigenic target genes [46, 47]. Moreover, NF-κB has been reported to enhance the transcriptional activity of YAP [48, 49]. Given the multifaceted chaperone functions of HSPA5, an intriguing question for future investigation is whether, in addition to stabilizing YAP/TAZ, HSPA5 indirectly modulates the stability or activity of other pivotal PMT regulators (e.g., STAT3, C/EBPβ) through its broader proteostatic functions, thereby further reinforcing the MES phenotype. Thus, the HSPA5–YAP/TAZ axis identified in our study likely functions as a central hub that cooperates with other signaling pathways to form a robust regulatory network sustaining tumor aggressiveness.

The Hippo pathway is a conserved signaling pathway that regulates cell proliferation during tissue development and regeneration, as well as organ size control and malignant progression in cancer [29, 50]. Dysregulation of the Hippo pathway promotes the nuclear translocation and hyperactivation of the transcriptional coactivators YAP and TAZ. This activation drives a YAP/TAZ-TEAD-mediated transcriptional program that induces cancer cell plasticity, drug resistance, and metastasis [30–32, 51]. However, recent evidence indicates that YAP/TAZ can also be activated through Hippo-independent mechanisms in human cancers [27, 34]. In our study, we demonstrated that overexpression of HSPA5 effectively prevented the serum starvation–induced degradation of YAP and TAZ, without altering their phosphorylation status, indicating that HSPA5 stabilizes YAP/TAZ independently of the canonical Hippo kinase cascade.

In addition, we found that HSPA5 knockdown significantly reduced YAP/TAZ protein levels without affecting their mRNA levels, suggesting that HSPA5 regulates YAP/TAZ at the post-transcriptional level. Evidence has shown that HSPA5 was also involved in maintaining protein stability in many human cancers [16–18]. Our findings showed that the proteasome inhibitor MG132 partially reversed the reduction in YAP/TAZ protein levels caused by HSPA5 deletion, and that HSPA5 overexpression prolonged the half-life of YAP/TAZ protein. These findings indicated that HSPA5 maintains YAP/TAZ protein stability by inhibiting proteasomal degradation. Among all the post-translational modifications, the ubiquitin-proteasome system (UPS) is the most important process that dynamically regulates protein stability [52]. The E3 ubiquitin ligase β-TrCP is a well-established regulator of YAP/TAZ, mediating their proteasomal degradation and cytoplasmic retention [34]. Our results showed that HSPA5 stabilizes YAP/TAZ by disrupting their interaction with β-TrCP, thereby preventing their ubiquitination and proteasomal degradation. Structurally, HSPA5 consists of an N-terminal nucleotide-binding domain (NDB) and a C-terminal substrate-binding domain (SBD) [53]. The SBD binds to substrates, while the NBD regulates the affinity of substrate binding to the SBD [54]. YAP and TAZ proteins share similar domains, including the TEAD transcription factor-binding domain (TBD), the transcriptional activation domain (TA), and the WW domain [32, 55]. The WW domain mediates protein-protein interactions and participates in various cellular processes in cancer [56]. Our results revealed that the SBD domain of HSPA5 was responsible for the interaction with the WW domain of YAP and TAZ, which was consistent with previous observations.

Previously, several small inhibitors of HSPA5 were identified, including YUM70, HA15, and EGCG [57–59]. For instance, EGCG binds to the ATPase catalytic domain (NBD) of HSPA5, thereby exerting potent anti-tumor activity [59]. HSPA5, a ubiquitously expressed chaperone essential for maintaining cellular homeostasis in normal cells, poses challenges for clinical application due to on-target toxicity. However, accumulating evidence indicates that tumor cells exhibit a heightened dependency on its chaperone activity compared with normal cells [36]. Therefore, the development of tumor specific delivery systems (such as nanoparticles) or selective targeting approaches represents a feasible and effective strategy for treating GBM. HSPA5 inhibition could potentially reverse the therapy-resistant phenotype associated with the MES state. We propose that a promising direction for future investigation is that HSPA5 inhibitors may synergize with temozolomide or radiotherapy by impairing DNA repair capacity and suppressing pro-survival signaling, thereby overcoming treatment resistance.

## Conclusions

In conclusion, we identified HSPA5 as a key regulator of YAP/TAZ, which promotes PMT and maintains MES characteristics in GBM. Mechanistically, HSPA5 stabilizes YAP/TAZ by disrupting their interaction with β-TrCP. These insights advanced our understanding of GBM pathogenesis and provided promising avenues for developing novel molecular targets and therapeutic strategies against this challenging disease.

### Limitations of the study

Our findings are primarily based on cell lines and xenograft models, which warrant future validation in more clinically relevant models, such as patient-derived organoids. Moreover, it remains essential to determine whether targeting HSPA5 can sensitize GBM to temozolomide or radiotherapy and to elucidate potential compensatory pathways for YAP/TAZ degradation in HSPA5-deficient cells.

## Supplementary information

Supplementary Figure Legends

Supplementary Figure 1

Supplementary Figure 2

Supplementary Figure 3

Supplementary Figure 4

Supplementary Figure 5

Supplementary Figure 6

Supplementary Figure 7

Supplementary Figure 8

Supplementary Table1

Supplementary Table2

Supplementary Table3

Supplementary Table4

Supplementary Table5

Supplementary Table6

Supplementary Table7

Original Western blot data

aj-checklist

## Data Availability

The datasets used and/or analyzed during the current study are available from the corresponding author on reasonable request.
