# Supplementary material for: HSPA5 promotes YAP/TAZ stability independently of the Hippo pathway and induces proneural-to-mesenchymal transition in glioblastoma
Source: Cell Death Dis. 2026 Feb 7;17(1):208. doi: 10.1038/s41419-026-08428-3 (PMC12895041; doi:10.1038/s41419-026-08428-3)
Supplement: Supplementary file 1 — Supplementary Figure Legends [file 41419_2026_8428_MOESM1_ESM.docx]

**HSPA5 promotes YAP/TAZ stability independently of the Hippo pathway and induces proneural-to-mesenchymal transition in glioblastoma**

**Figure. S1** HSPA5 is highly expressed in GBM and is correlated with MES subtype.

**A-B** WB analysis of HSPA5 (A) and HSPA6 (B) protein levels in normal brain tissues (NBTs, n=3), proneural (PN, n=3), classical (CL, n=3), and mesenchymal (MES, n=3) GBM patient samples. GAPDH serves as a loading control.

**C** Representative immunohistochemical (IHC) staining images of HSPA6 in NBTs, PN, CL, and MES GBM samples. Scale bar: 50 μm.

**Figure. S2** HSPA5 may be involved in regulating PMT.

**A** microscopy images showing the distinct morphological features of primary proneural (PN20, PN24) and mesenchymal (MES50, MES52) GBM cells in culture. Scale bar: 200 μm.

**B** qRT-PCR analysis of *HSPA5* mRNA levels in normal human astrocytes (NHA), PN (PN20, PN24), and MES (MES50, MES52) cells.

**C** qRT-PCR analysis of *HSPA5* mRNA level in MES cells transduced with shNC and shHSPA5.

**D** qRT-PCR analysis of *HSPA5* mRNA level in PN cells transduced with Vector and HSPA5.

**E-F** Transwell migration assay was used to analyze the migratory ability of MES cells transduced with shNC and shHSPA5.

**G-H** Transwell migration assay was used to analyze the migratory ability of PN cells transduced with Vector and HSPA5.

**I-J** Transwell invasion assay was used to analyze the invasive ability of MES cells transduced with shNC and shHSPA5.

**K-L** Transwell migration assay was used to analyze the invasive ability of PN cells transduced with Vector and HSPA5.

Data were normalized to GAPDH and are presented as mean ± SD. *P < 0.05, **P < 0.01 and ***P < 0.001. Two‐tailed unpaired t‐test (B, C, and D).

**Figure. S3** HSPA5 facilitates PMT in GBM by modulating YAP/TAZ.

**A** GSEA based on the Verhaak gene set showed that *HSPA5* knockdown in MES50 cells was negatively correlated with the MES subtype and positively correlated with the PN subtype.

**B** ssGSEA showing the enrichment scores for the proneural and mesenchymal gene signatures in MES50-shNC and MES50-shHSPA5 cells based on transcriptomic data.

**C** qRT-PCR and WB analysis of HSPA5 expression levels in MES cells transduced with shNC and shYAP/TAZ.

**D-E** Colony formation (D) and CCK8 (E) assays were used to evaluate cell proliferation ability in shNC/shHSPA5 MES cells treated with indicated interventions (transfection of unphosphorylatable YAP-5SA/TAZ-4SA).

**F-G** Transwell assay was used to assess cell migratory (F) and invasive (G) ability in shNC/shHSPA5 MES cells treated with indicated interventions (transfection of unphosphorylatable YAP-5SA/TAZ-4SA).

**H-I** Colony formation (H) and CCK8 (I) assays were used to evaluate cell proliferation ability in Vector/HSPA5 PN cells treated with indicated interventions (knockdown of YAP/TAZ).

**J-K** Transwell assay was used to assess cell migratory (J) and invasive (K) ability in Vector/HSPA5 PN cells treated with indicated interventions (knockdown of YAP/TAZ).

Data are shown as mean ± SD. *P < 0.05, **P < 0.01 and ***P < 0.001. Two‐tailed unpaired t‐test (B-J).

**Figure. S4** CD44 and c-MET are regulated by YAP/TAZ at the transcriptional level.

**A-B** qRT-PCR analysis of *YAP1*, *WWTR1*, *CD44*, and *c-MET* mRNA level in MES cells transduced with shNC and shYAP/TAZ.

**C** WB analysis of YAP, TAZ, CD44, and c-MET protein levels in MES cells transduced with shNC and shYAP/TAZ.

**D-E** The mRNA expression levels of TEAD family transcription factors in TCGA GBM datasets and the Gulou GBM datasets.

**F-G** The publicly available ChIP-seq data showed that TEAD1 binds to the promoter regions of CD44 and c-MET.

**H** A schematic representation was developed to illustrate the predicted TEAD1 binding sequences within the promoter region of CD44 and c-MET.

**I-J** Chromatin immunoprecipitation quantitative PCR (ChIP-qPCR) analysis of TEAD1 binding to specific regions of the CD44 (I) and c-MET (J) promoters in MES50 cells.

**K-L** Dual-luciferase reporter assays measuring the activity of wild-type (WT) or TEAD1-binding-site mutant (Mut) CD44 (K) and c-MET (L) promoters in HEK293T cells expressing Vector or constitutive active YAP-5SA/TAZ-4SA. Firefly luciferase activity was normalized to Renilla.

Data are shown as mean ± SD. *P < 0.05, **P < 0.01 and ***P < 0.001. Two‐tailed unpaired t‐test (A, B, and I-L).

**Figure. S5** HSPA5 stabilizes YAP/TAZ independent of the Hippo pathway.

**A-C** Spearman correlation analysis between *HSPA5* and *YAP1* or *WWTR1* mRNA expression levels in TCGA datasets for bladder carcinoma (BLCA, A), liver hepatocellular carcinoma (LIHC, B), and lung adenocarcinoma (LUAD, C).

**D-F** WB analysis of YAP and TAZ protein levels in BLCA (T24, D), LIHC (HepG2, E), and LUAD (A549, F) cell lines transduced with shNC and shHSPA5.

**G** Quantification of cytoplasmic and nuclear YAP and TAZ protein levels from the subcellular fractionation experiment shown in Figure 4C.

**H** Immunofluorescence staining was performed to examine the subcellular localization of YAP and TAZ in PN20 and PN24 cells.

Data are shown as mean ± SD. *P < 0.05, **P < 0.01 and ***P < 0.001. Two‐tailed unpaired t‐test (G).

**I** WB analysis of YAP/TAZ in control (shNC) and MST1/2 double-knockdown (shMST1/2) MES50 cells with or without HSPA5 knockdown.

**Figure. S6** HSPA5 SBD domain stabilizes YAP/TAZ by disrupting its interaction with β-TrCP.

**A** CTGF luciferase reporter assay in HEK293T cells co-transfected with FLAG-YAP, FLAG-TAZ, HA-β-TrCP, and HSPA5 expression vectors as indicated. Firefly luciferase activity was normalized to Renilla.

**B** qRT-PCR analysis of *β-TrCP* knockdown efficiency in MES cells transfected with siNC and siβ-TrCP siRNA siRNA.

**C** WB analysis of HSPA5, β-TrCP, YAP and TAZ protein levels in HSPA5-depletion MES52 cells and transfected with siNC and siβ-TrCP siRNA.

**D** CTGF luciferase reporter activity in HEK293T cells stably expressing shNC or shHSPA5 and transfected with siNC or siβ-TrCP siRNA.

**E** WB analysis of CD44 and c-MET protein levels in HSPA5-depletion MES cells and transfected with siNC and siβ-TrCP siRNA.

**F-G** qRT-PCR analysis of *CD44* (F) and *c-MET* (G) mRNA levels in HSPA5-depletion MES cells and transfected with siNC and siβ-TrCP siRNA.

**H-I** Colony formation (H) and CCK8 (I) assays were used to evaluate cell proliferation ability in shNC/shHSPA5 MES cells transfected with siNC and siβ-TrCP siRNA.

**J-K** Transwell assay was used to assess cell migratory (J) and invasive (K) ability in shNC/shHSPA5 MES cells transfected with siNC and siβ-TrCP siRNA.

Data are shown as mean ± SD. *P < 0.05, **P < 0.01 and ***P < 0.001. Two‐tailed unpaired t‐test (A, B, D, F-K).

**Figure. S7** HSPA5 interacts with YAP/TAZ.

**A-D** GST pull-down assay was performed to detect the interaction of HSPA5 and YAP or TAZ.

**E** Proximity ligation assay (PLA) assay of HSPA5-YAP/TAZ expression in GBM cells using anti-HSPA5, anti-YAP, and anti-TAZ antibodies.

**F** MES50 cells were transfected with the HA-HSPA5, Myc-YAP, and Flag-TAZ plasmids. After 48 h transfection, an immunofluorescence assay was conducted to acquire the images by using fluorescence microscopy.

**Figure. S8** HSPA5 SBD domain stabilizes YAP/TAZ by disrupting its interaction with β-TrCP.

**A** Endogenous Co-IP assays were performed in MES50-shNC and MES50-shLATS1/2 cells following MG132 (20 µM for 8 h) treatment. The interaction between HSPA5 and YAP or TAZ were subsequently examined.

**B** Molecular docking model of HSPA5 with YAP domains identified THR460, THR462, THR428, GLU427, LYS464, and ASN467 as potential binding sites. Docking model of HSPA5 with TAZ domains identified GLY430, LEU472, and LYS472 as potential binding residues.

**C** WB analysis of YAP and TAZ protein levels in PN20 cells transfected with full-length and truncated HSPA5 constructs**.**

**D** 8xGTIIC-Luc activity in HEK293T cells co-transfected with YAP or TAZ and the full-length and truncated HSPA5 constructs (HA-Vector, HA-HSPA5 FL, HA-HSPA5 ΔNBD, and HSPA5 ΔSBD).

**E** qRT-PCR analysis of *CTGF* and *CYR61* mRNA levels transfected with full-length and truncated HSPA5 constructs (HA-Vector, HA-HSPA5 FL, HA-HSPA5 ΔNBD, and HSPA5 ΔSBD).

**F-G** CCK8 (J) and colony formation (K) assays were used to evaluate cell proliferation ability in PN20 cells transfected with full-length and truncated HSPA5 constructs (HA-Vector, HA-HSPA5 FL, HA-HSPA5 ΔNBD, and HSPA5 ΔSBD).

**H** Transwell assay was used to assess cell migratory and invasive ability in PN20 cells transfected with full-length and truncated HSPA5 constructs (HA-Vector, HA-HSPA5 FL, HA-HSPA5 ΔNBD, and HSPA5 ΔSBD).

**I-J** WB analysis of His-β-TrCP immunoprecipitates performed with lysates prepared from cells transfected with increasing amounts of HA-HSPA5.

**K** Immunohistochemical staining was used to examine the expression levels of N-cadherin and C/EBPβ in xenograft tumor tissues from different experimental groups. Scale bar: 50 μm.

Data are shown as mean ± SD. *P < 0.05, **P < 0.01 and ***P < 0.001. Two‐tailed unpaired t‐test (H-L).
