# Supplementary figures and images for: HSPA5 promotes YAP/TAZ stability independently of the Hippo pathway and induces proneural-to-mesenchymal transition in glioblastoma
Source: Cell Death Dis. 2026 Feb 7;17(1):208. doi: 10.1038/s41419-026-08428-3 (PMC12895041; doi:10.1038/s41419-026-08428-3)

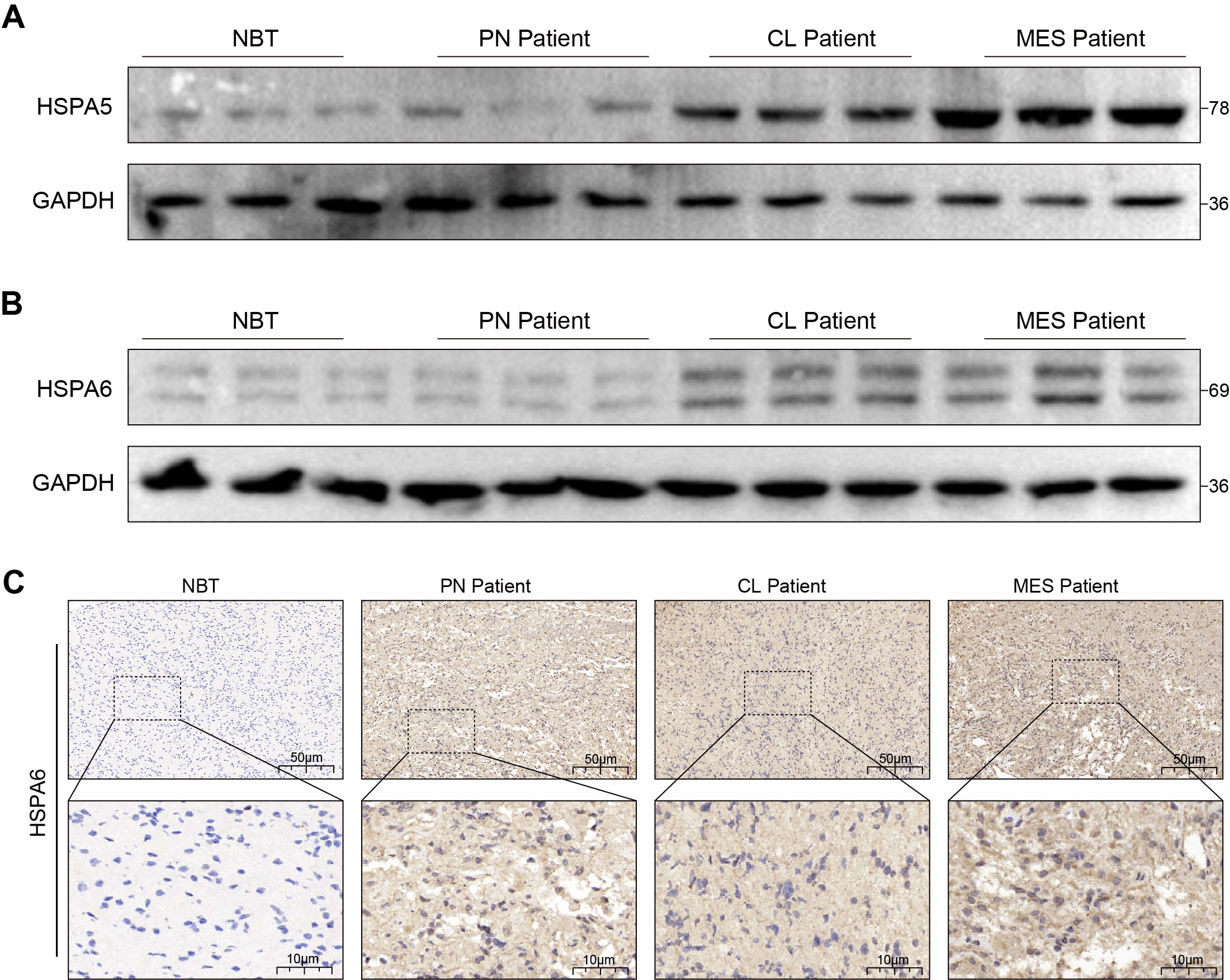

Supplement: Supplementary file 2 — Supplementary Figure 1 [file 41419_2026_8428_MOESM2_ESM.jpg]

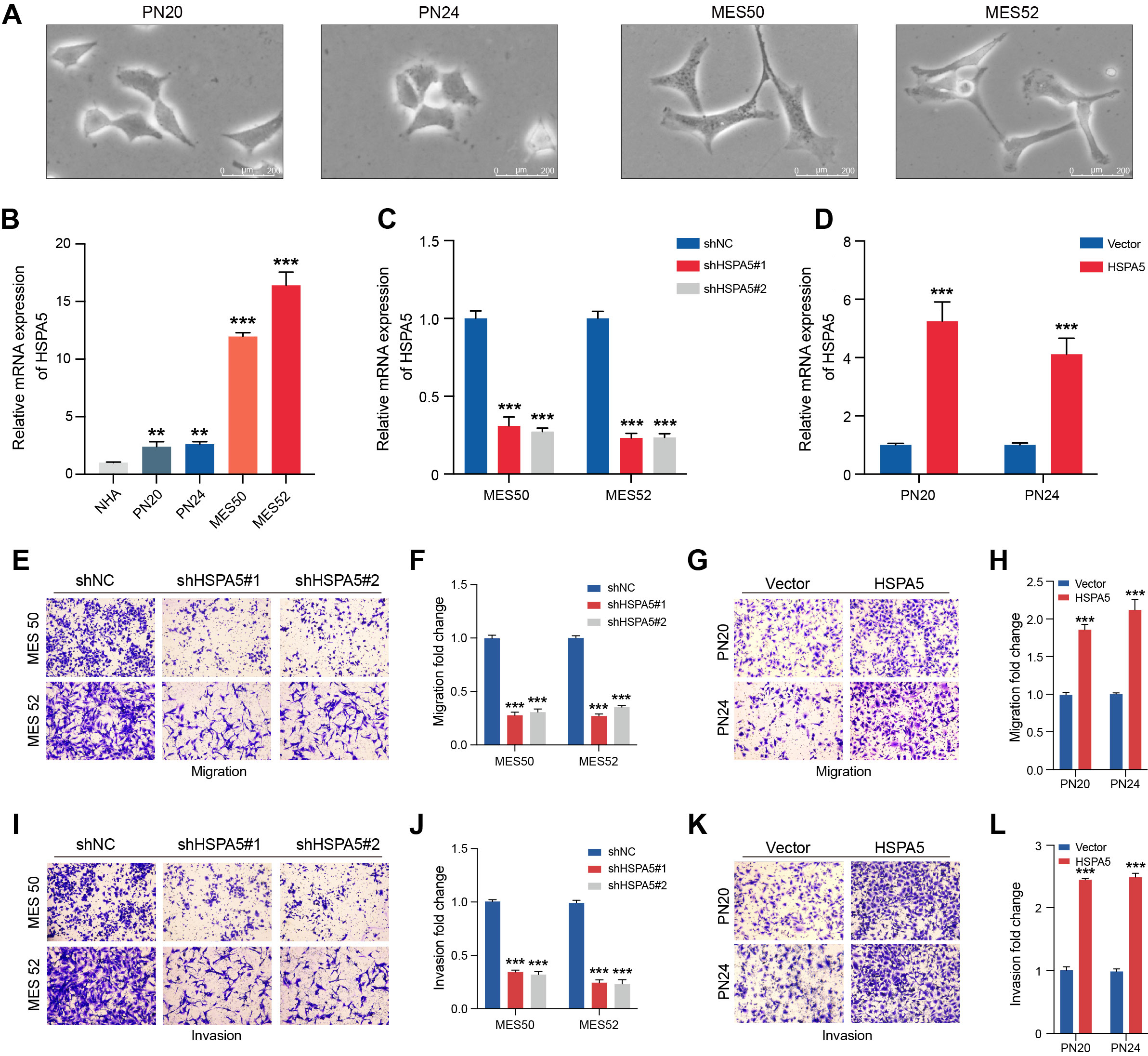

Supplement: Supplementary file 3 — Supplementary Figure 2 [file 41419_2026_8428_MOESM3_ESM.jpg]

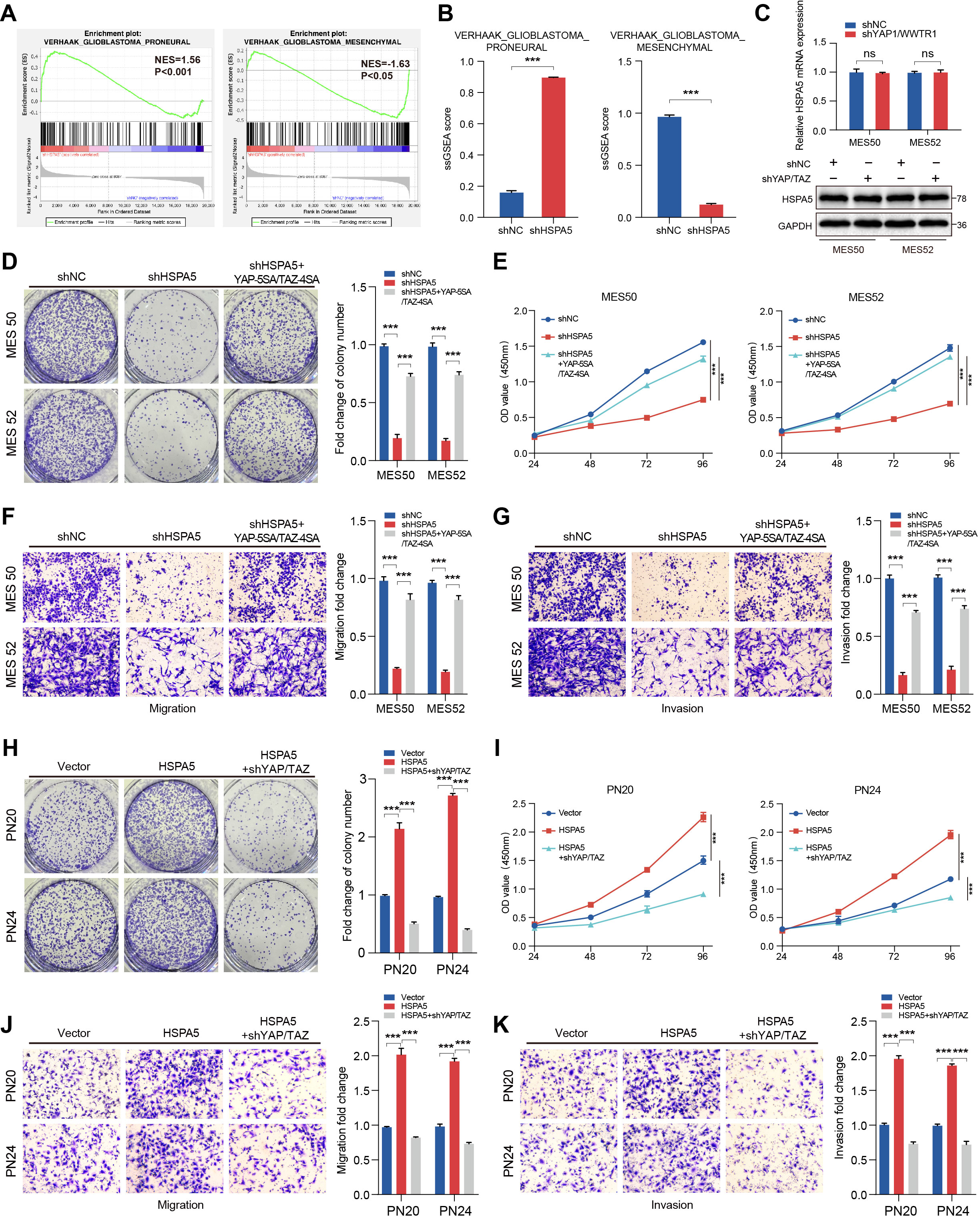

Supplement: Supplementary file 4 — Supplementary Figure 3 [file 41419_2026_8428_MOESM4_ESM.jpg]

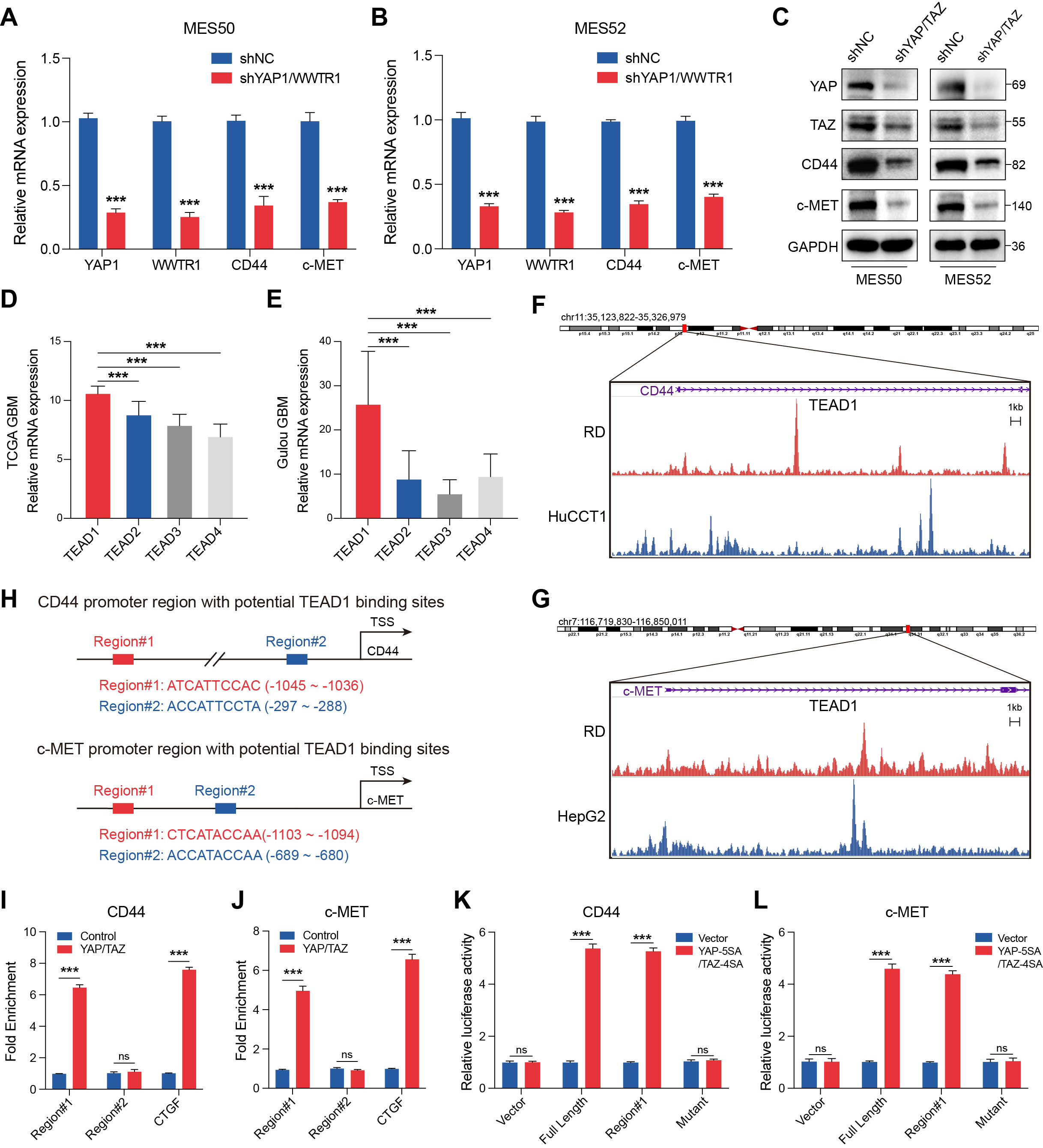

Supplement: Supplementary file 5 — Supplementary Figure 4 [file 41419_2026_8428_MOESM5_ESM.jpg]

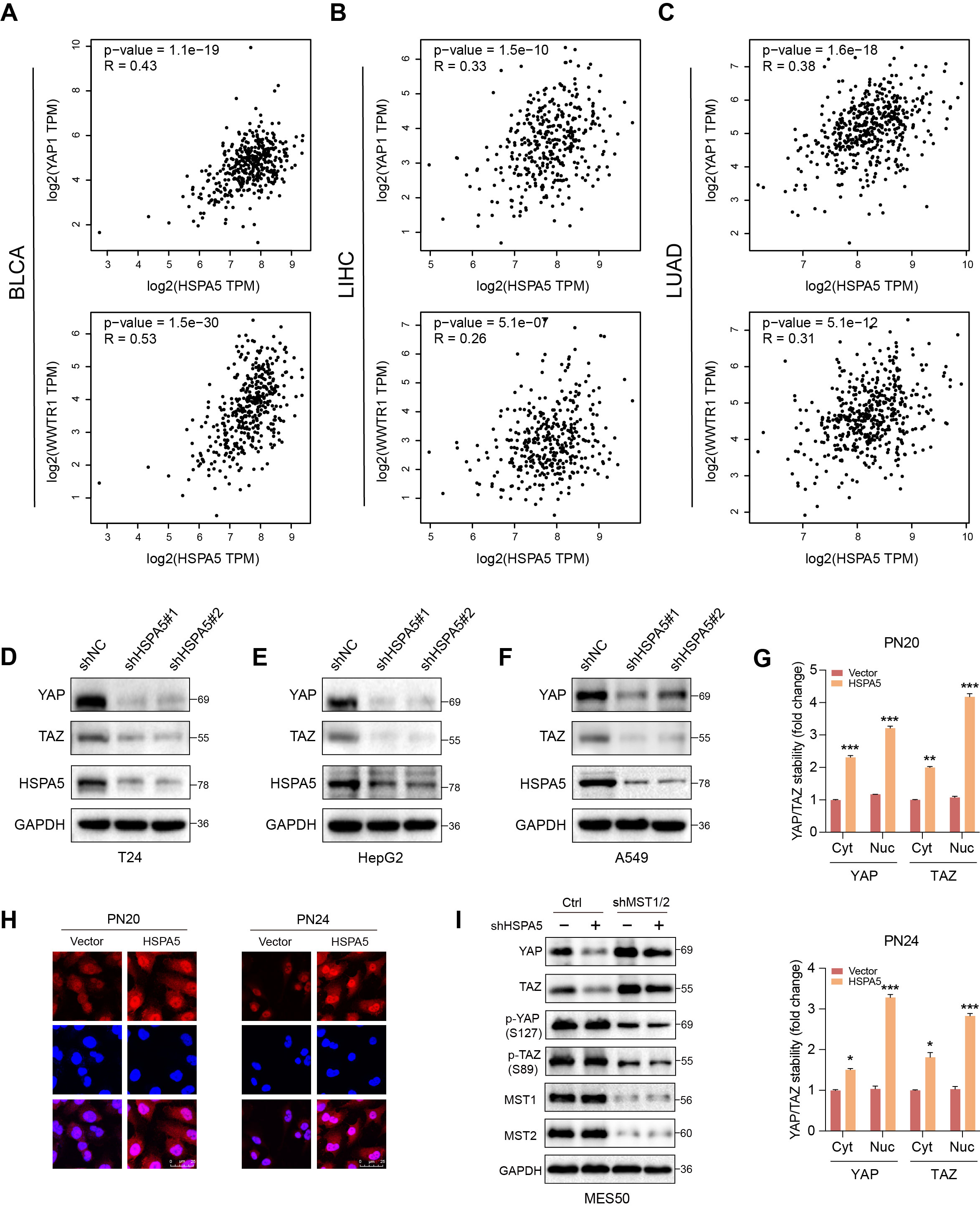

Supplement: Supplementary file 6 — Supplementary Figure 5 [file 41419_2026_8428_MOESM6_ESM.jpg]

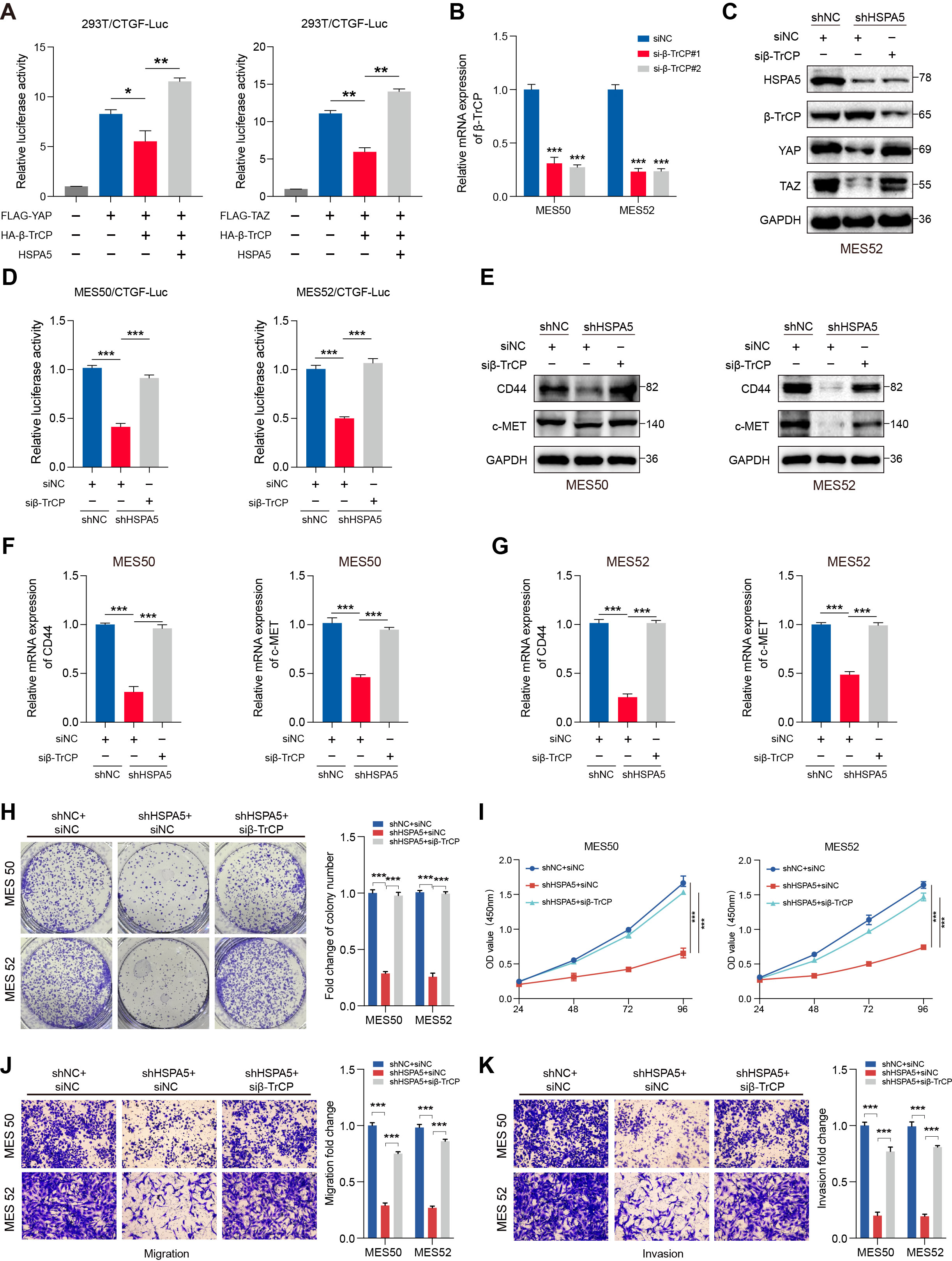

Supplement: Supplementary file 7 — Supplementary Figure 6 [file 41419_2026_8428_MOESM7_ESM.jpg]

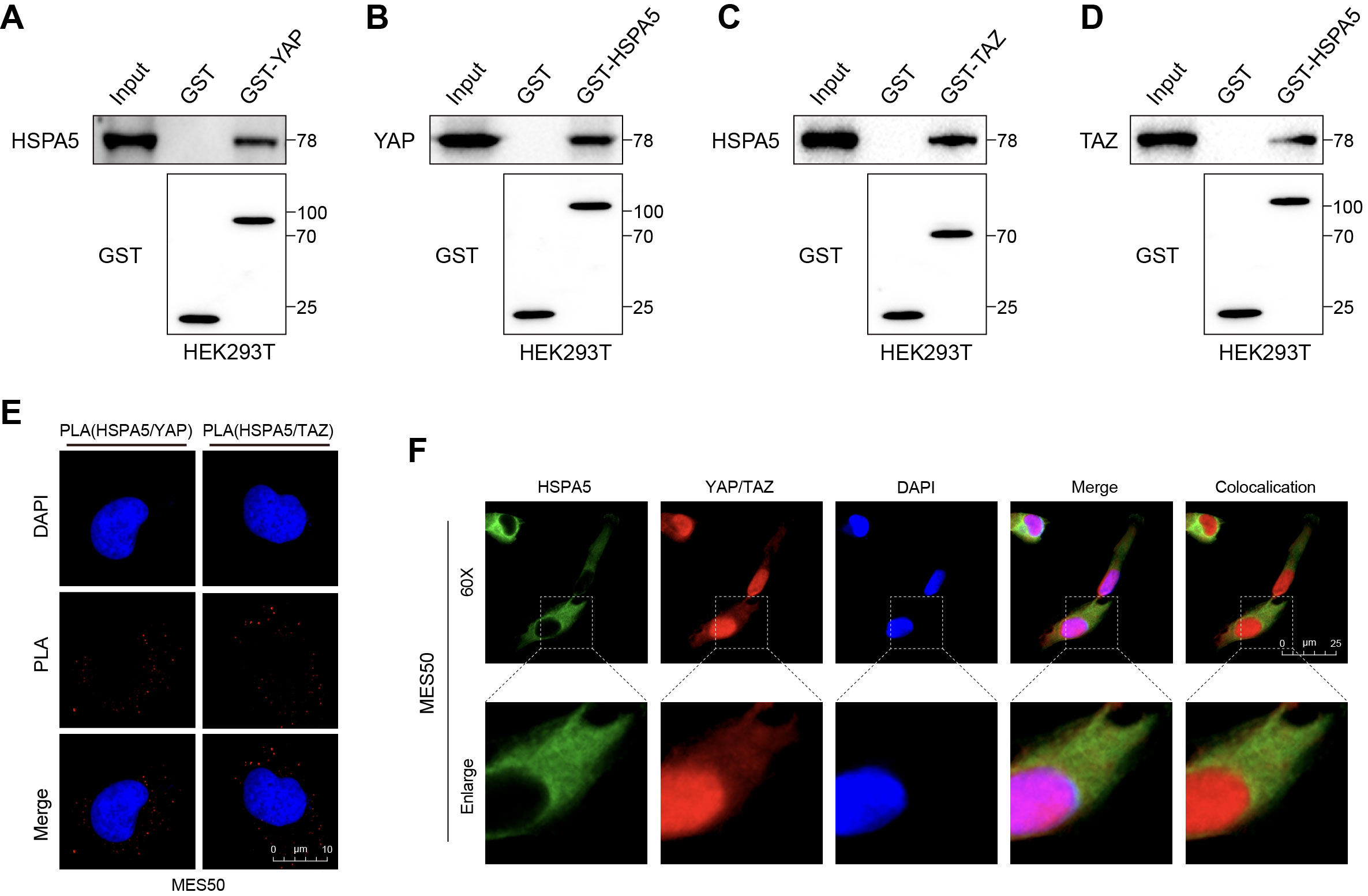

Supplement: Supplementary file 8 — Supplementary Figure 7 [file 41419_2026_8428_MOESM8_ESM.jpg]

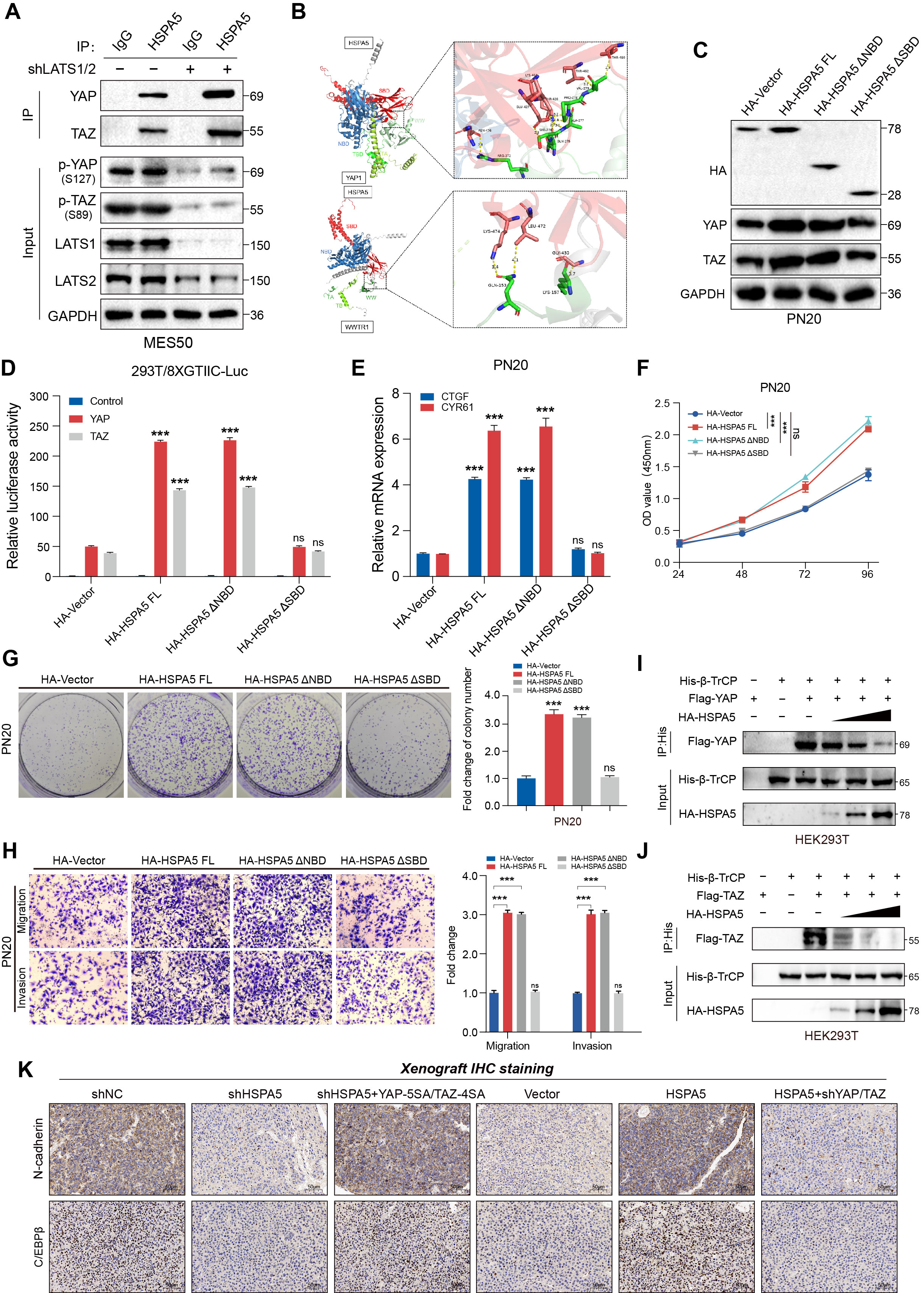

Supplement: Supplementary file 9 — Supplementary Figure 8 [file 41419_2026_8428_MOESM9_ESM.jpg]

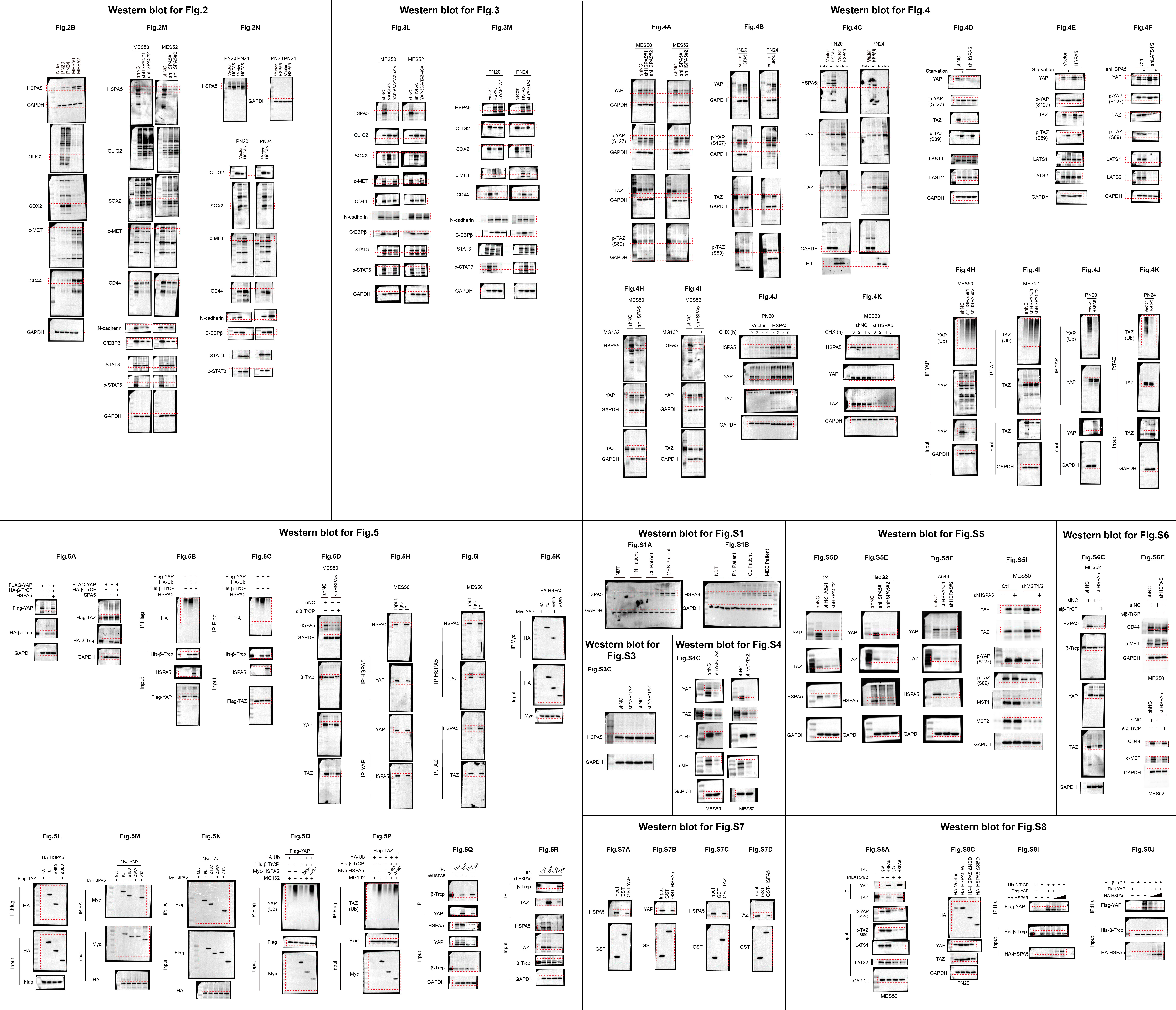

Supplement: Supplementary file 17 — Original Western blot data [file 41419_2026_8428_MOESM17_ESM.jpg]
